# Relationship Between the Fatty Acid Profiles and Gut Bacterial Communities of the Chinese Mitten Crab (*Eriocheir sinensis*) From Ecologically Different Habitats

**DOI:** 10.3389/fmicb.2020.565267

**Published:** 2020-10-15

**Authors:** Shengyan Su, Brian Pelekelo Munganga, Fukuan Du, Juhua Yu, Jianlin Li, Fan Yu, Meiyao Wang, Xinjin He, Xinyuan Li, Raouf Bouzoualegh, Pao Xu, Yongkai Tang

**Affiliations:** ^1^Key Laboratory of Genetic Breeding and Aquaculture Biology of Freshwater Fishes, Ministry of Agriculture, Freshwater Fisheries Research Center, Chinese Academy of Fishery Sciences, Wuxi, China; ^2^Wuxi Fisheries College, Nanjing Agricultural University, Wuxi, China

**Keywords:** Chinese mitten crab, fatty acid profiles, gut bacterial community, geographic location, fatty acids and gut microbial interactions

## Abstract

The gut microbiota plays an important role in a variety of physiological functions such as intestinal digestion, metabolic homeostasis, immune response, and responses to disease treatment. Whether there is a relationship between gut microbial communities and fatty acid (FA) profiles of Chinese mitten crab is unclear. Hence, we analyzed the relationship between FA profiles and the gut bacterial communities of six Chinese mitten crab (*Eriocheir sinensis*) populations from different lakes. The crabs were sampled from six different lakes in Jiangsu Province, China. The FA profiles of these crab populations were compared and clustered, and then used to determine the relationship between geographic location and FA composition. We also characterized the gut microbial communities of these crabs using 16S rRNA high-throughput gene sequencing. The FA profiles varied significantly (*P* < 0.05) between crabs from different geographical locations. A similar trend was also observed in the gut microbial communities, which also varied significantly based on their geographical origin (*P* < 0.05). Furthermore, alpha diversity, cluster analysis, and matching bacterial community structures with specific locations revealed patterns that significantly linked FA profiles to the gut microbiota. Further analysis of FA profiles and gut microbial community generated patterns that linked the two parameters. Hence, it was observed that the gut microbial community seems to contribute significantly to the FA composition of the Chinese mitten crab. However, further studies need to be conducted to investigate the interactions between gut microbial communities and the biochemical composition of the Chinese mitten crab, which will ultimately unravel the complexity of microbial ecosystems for potential applications in aquaculture and species conservation.

## Introduction

The Chinese mitten crab (*Eriocheir sinensis*) is one of the most economically important and nutritious crustaceans in China (Chen et al., 2007). It is a medium-sized, freshwater burrowing crab native to eastern coastal rivers and estuaries that feeds into the Yellow Sea and spans both China and Korea (Zhang et al., 2001; Rudnick et al., 2003). It spends most of its life in freshwater, and then migrates to the sea or ocean to reproduce (a catadromous species) (Zhang et al., 2001; Rudnick et al., 2003; Cheng et al., 2008; Sui et al., 2009). The Chinese mitten crab production and value greatly expanded between 1990 and 2012, which reflects the increasing importance of the species to China’s aquaculture sector (Chen et al., 2007; Chen et al., 2015; Wang et al., 2015, 2016). In 2015, approximately 820,000 tons of these crabs were harvested, which accounted for more than ¥50 billion in value (People’s Republic of China Ministry of Agriculture, 2016; Dong et al., 2018). However, there are some challenges and limitations associated with its culture (e.g., potential disease outbreak), conservation, and management; and the need to improve its nutritional value (Ding et al., 2016; Shen et al., 2017). Consequently, more attention has been given to understanding its health, nutrition, and conservation.

A recent research approach to improving the health, nutrition, and economic values and the conservation of aquatic species is to understand the role of gut microbial communities (Roeselers et al., 2011; Egerton et al., 2018; Zeng et al., 2018; Trevelline et al., 2019). Recent studies have shown that microbial communities play significant roles in animal intestinal digestion, immune response, physiology, and response to disease treatment (Heijtz et al., 2011; Stanley et al., 2014; Waite and Taylor, 2015; Zheng et al., 2017; Ramos and Hemann, 2017; Byndloss et al., 2017). Along this line of thought, some studies have been conducted to understand the principles governing microbial community assembly and maintenance within the gut of the Chinese mitten crab by developing robust model systems to study host-microbial interactions (Chen et al., 2015; Zhang et al., 2016; Dong et al., 2018). A recent study examined the bacterial communities found in the gut of crabs cultured in Lake Tai, China (Chen et al., 2015). In another study, Zhang et al. (2016) assessed the differences between microbiota found in the gut and those found in the surrounding environment. Furthermore, Dong et al. (2018) conducted a comparative gene expression analysis of the intestinal bacterial community and the expression of gut immunity genes in these crabs. However, enormous knowledge gaps still exist regarding the interaction between the gut microbial community and the biochemical composition of these crabs.

In general, no links have been established between gut microbial communities and the proximate composition of the Chinese mitten crab. However, previous studies assessing either the relationship between the proximate composition of animals and geographic location or microbial communities in animals and their locality suggest possible links between the gut microbial community and proximate composition (Zenebe et al., 1998; Riveiro et al., 2011). The proximate composition of the same species living in different environments has been reported to be significantly different (Zenebe et al., 1998; Riveiro et al., 2011; Roeselers et al., 2011; Mohan, 2013, p. 960–963; Antony, 2016). Likewise, the quantity and composition of the gut microbiota of host species have been shown to vary significantly based on their geographic location (Finkel et al., 2011; Franchini et al., 2014). Furthermore, some studies have shown direct links between gut microbiota and proximate composition. For example, in quails and humans, modulation of the gut microbiota induced changes in fatty acid (FA) profiles. In laying Japanese quails (*Coturnix coturnix japonica*), the FA composition of liver lipids can be modified by modulating the gut microbiota (Furuse et al., 1992). Moreover, some polyunsaturated fatty acids (PUFA) derived bacterial metabolites were identified, which were correlated with specific fecal bacteria (*Bifidobacterium* species, *Eubacterium ventriosum*, and *Lactobacillus* species) (Druart et al., 2014).

Understanding gut microbiota composition, abundance, and related environmental factors can provide insights to support the effective management, conservation, and improvement of the economic performance of the crab industry. For example, Zhang et al. (2019) identified an effective mechanism present in the environment that can be utilized to improve a species’ growth, which in turn can improve its economic performance. By combining genetics, microbiota, and growth performance, NRT1.1B was found to be a link between root microbiota composition and nitrogen use in rice agriculture (Zhang et al., 2019).

The aim of this study was to establish the relationship between FA profiles and the gut bacterial community of the Chinese mitten crab from ecologically different habitats. For this purpose, we analyzed the FA profiles and gut microbiota of six crab populations sampled from six different lakes in China’s Jiangsu province. The FA profiles of these crab populations were compared and clustered, and then used to determine the relationship between geographic location and FA profiles. Furthermore, we characterized the microbial communities from the gut content of the crabs using 16S rRNA high-throughput gene sequencing. Alpha diversity, cluster analysis, and the bacterial community structures in specific locations were observed to determine their contribution to the FA profiles.

## Materials and Methods

### Sample Collection

**FIGURE 1:**
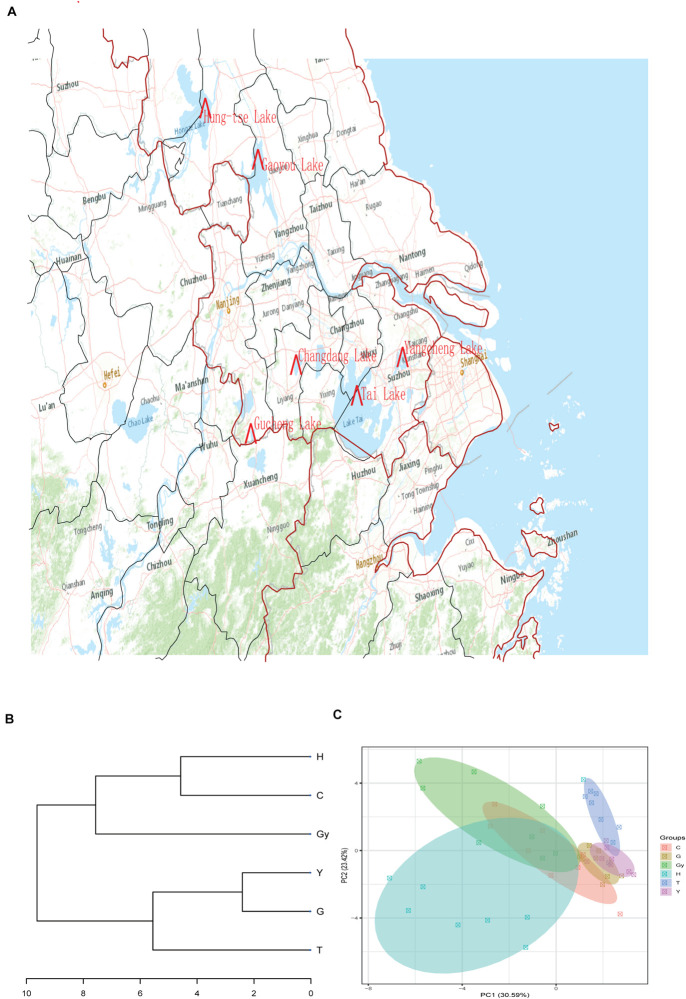
Sample collection sites, dendrogram cluster, and PCA. **(A)** Sample collection sites. **(B)** Dendrogram cluster analysis of the crab populations based on their fatty acid profiles. **(C)** PCA analysis of the crab populations based on their fatty acid profiles.

In the present study, six populations of the Chinese mitten crab (*Eriocheir sinensis*) weighing – 120–150 g were obtained from six different lakes in China’s Jiangsu province in December 2018. The lakes are Changdang Lake (C), Gucheng Lake (G), Gaoyou Lake (Gy), Hung-tse Lake (H), Taihu Lake (T), and Yangcheng Lake (Y) (Figure 1A and Table 1). These lakes are part of the Yangtze River drainage basin system, they form an indirect but continuous water system with Yangtze River. The Chinese mitten crab populations migrate from the six lakes into the Yangtze River estuary to spawn. A total of 180 crabs were collected, 30 from each lake (15 males and 15 females) and brought to the Freshwater Fisheries Research Center of the Chinese Academy of Fishery Sciences. Five sampling sites were purposely selected in each lake. The biochemical parameters for different lakes were not analyzed, nonetheless, the standard water parameters for the lakes where the crab populations were sampled can be found at (GB3838-2002). Upon arrival, a total of 48 crabs were randomly selected from all the populations (eight crabs from each lake, four males and four females).

**TABLE 1:** Sampling site information.

Site	Name	Habit	Area (km^2^)	Coordinate		N
C	CHANGDANG HU	Lake	89	N31°59′–31°62′	E119°52′–118°60′	8
G	GUCHENG HU	Lake	65	N31°14′–31°18′	E118°53′–118°57′	8
Gy	GAOYOU HU	Lake	674.7	N32°42′–33°41′	E119°06′–119°25′	8
H	HONGZE HU	Lake	1576.9	N33°06′–33°40′	E118°10′–118°52′	8
T	TAI HU	Lake	2425	N30°55′40″–31°	E119°52′32″–120°	8
Y	YANGCHENG HU	Lake	119.04	N30°55′40″–30°	E119°51′32″–120°	8

### Sample Preparation

#### Gut Microbial Sample

The body surfaces of the randomly selected crabs (48) were washed with sterile water and disinfected with 75% ethanol for 2 min. They were subsequently dissected to remove the digestive tract to collect the gut contents (distal section). The gut contents were collected into sterile tubes and stored in a −80°C freezer and were subsequently used for bacterial DNA extraction. The rest of the crab body parts that remained after collecting gut contents were also stored at −80°C.

#### FA Sample

The 48 crabs were collected from the freezer 2 days from the day of arrival and allowed to thaw, and then 6–8g of muscle was removed from the appendage carapaces of each crab. These muscle tissues were dried using a vacuum freeze dryer (Labconco Corp., Kansas City, MO, United States) and grounded into powder form with a mortar and pestle. The dried samples were kept in ziplock plastic bags and stored at −20. Thereafter, they were sent to the lab for FA profile analysis.

### Fatty Acid Extraction and Analysis

Approximately 2–3g of the ground muscle tissues from each crab were added into tubes and a mixed solution (chloroform/methanol, 2:1 v/v) was added and oscillatory extraction was conducted. Oscillatory extraction was done three times, followed by filtering, and evaporation drying of the filtrates. Thereafter, 2 mL of 0.5 mol/L sodium hydroxide in methanol was added, and the solution was put in a water bath at 60°C for 30 min. The solution was allowed to cool, then 2 mL of 25% boron trifluoride in methanol was added and put in a water bath again at 60°C for 20 min. After cooling, 2 mL of n-hexane and 2 mL of saturated sodium chloride solution were added to obtain fatty acid methyl esters. The fatty acid methyl esters obtained were analyzed by gas chromatography (Chromatographic column: DB-WAX 30 M I. D. 0.32 mm; Shimadzu GC-2030, Europe). The temperature of the column was initially held at 100°C for 3 min; then increased to 180°C at a rate of 10°C/min and held for 1 min; and finally increased to 240°C at a rate of 3°C/min and held for 15 min. The whole analysis process took 95 min. The carrier gas was Nitrogen (N_2_) with a flow rate of 3 mL/min, fuel gas was hydrogen (H_2_) with a flow rate 40 mL/min and oxidant gas was air with a flow rate 400 mL/min. The injection port and the temperature detector were kept at 250°C. Identification of the FA was based on the known standard time of retention (Sigma-Aldrich Co., St. Louis, MO, United States). FA composition are presented as the percentage of each FA compared to the total fatty acids.

### DNA Extraction

Prior to DNA extraction, the intestinal contents and mucosa from each individual crab were collected and mixed completely using a hand-held tissue homogenizer. Each sample of intestinal contents and mucosa from 48 crabs were divided into 14 tubes. The genomic DNA of the sample was extracted using commercially available kit (E.Z.N.A® Genomic DNA Isolation Kits, Omega Bio-Tek), following the kit manufacturer protocols. The appropriate amount (50 μL) of sample DNA was collected into the centrifuge tube. Then the purity and concentration of DNA tested by electrophoresis (1% agrose gel, 5μL Nared (dye), 4 μl of DNA sample, 2 μL of loading buffer and 4 μL of DL-1000 maker). The samples were stored at −20°C and were later sent for further analysis.

### Polymerase Chain Reaction (PCR) Amplification, Purification, and Pyrosequencing

Based on the genomic region of DNA, specific primers with barcodes were selected according to the selection of the sequencing region. Universal polymerase chain reaction (PCR) primers 515F (5′-GTGCCAGCMGCCGCGGTAA-3) and 806R (5′-GGACTACHVGGGTWTCTAAT-3) (Magoc and Salzberg, 2011; Caporaso et al., 2012), targeting the V4 hypervariable region of the 16S rRNA gene (400–450 bp) were used to identify bacterial diversity. PCR reactions were performed using the Phusion® High-Fidelity PCR Master Mix (New England Biolabs, Ipswich, United States) according to the manufacturer’s instructions. Samples were amplified in triplicate to a total reaction volume of 50 μL. Each 50 μL reaction mixture contained 25 μL Phusion® High-Fidelity PCR Master Mix (New England Biolabs, Ipswich, United States), 2.5 μL of each forward and reverse primer (10 pmol each), 1 μL DNA template (∼5 ng), and 19 μL nuclease-free water. The thermocycling conditions were as follows: initial denaturing, 98°C for 30 s, followed by 35 cycles of 10 s at 98°C (denaturing), 30 s at 65°C (annealing), 30 s at 72°C (extension), and a final extension for 10 min at 72°C. The amplicons were evaluated on 2% agarose gels. The PCR products were mixed with equal volumes of 1X loading buffer containing SYBR Green prior to loading onto the gels. PCR products were then purified with a Qiagen Gel Extraction Kit (Qiagen, Hilden, Germany) according to the manufacturer’s instructions. Sequencing libraries were created using the TruSeq® DNA PCR-Free Sample Preparation Kit (Illumina, San Diego, United States) following the manufacturer’s guidelines, and index codes were added. DNA was quantified using a Qubit® 2.0 fluorometer with the aid of a Qubit® dsDNA HS Assay (Thermo Fisher Scientific, Waltham, United States) and the Agilent Bioanalyzer 2100 system. Sequencing was carried out on the Illumina HiSeq 2500 platform, and 250 bp paired-end reads were generated.

### Statistical Analysis

Sequence analysis [including operational taxonomic unit (OTU) identification], taxonomic allocations, and evaluation of community composition were primarily conducted with the MOTHOR software (Kozich et al., 2013). Paired-end reads were allocated to samples based on their specific barcodes and shortened by trimming off the barcode and primer sequence. Paired-end reads were merged using FLASH (V1.2.7)^1^ (Magoc and Salzberg, 2011). In order to obtain high-quality clean tags, the raw tags were subjected to quality control processes using the QIIME program (V1.7.0)^2^. Chimeric sequences were identified and removed using the UCHIME algorithm (UCHIME Algorithm)^3^ to obtain effective tags. The sequences were analyzed using the Uparse software (Uparse v7.0.1001)^4^, and sequences with ≥ 97% similarity were allocated to the same OTUs. For annotation, representative sequences for each OTU were screened. For each representative sequence, the GreenGene Database^5^ based on the Ribosomal Database Project (RDP) classifier (Version 2.2)^6^ algorithm was used to annotate taxonomic information. To investigate the phylogenetic relationship of OTUs, and the differences between dominant bacterial species in the crab population (groups), multiple sequence alignments were performed using the MUSCLE software (Version 3.8.31)^7^. Principal coordinate analysis (PCoA) was conducted to obtain principal coordinates and visualize the complex, multidimensional data. The weighted correlation network analysis (WGCNA), stat, and ggplot2 packages in the R software (Version 2.15.3) were used to display the PCoA analysis. Identified OTUs, abundance-based coverage estimator (ACE), Shannon, Simpson, Goods-coverage, and Chao1 were calculated with QIIME (Version 1.7.0) and visualized using the R software (Version 2.15.3). Rarefaction analysis was performed for all five libraries, and the heatmaps, Venn diagrams, and species rank abundance distribution curves were generated using the R Project for statistical computing^8^. The distances between gut microbial communities in the six crab populations were calculated using the weighted UniFrac beta-diversity metric through QIIME. Non-metric multidimensional scaling (NMDS) was used to visualize the pairwise UniFrac distances among samples. Statistical analysis was also carried out using the Kruskal-Wallis test.

The FA profile data of crab populations obtained was subjected to multivariate ANOVA using the R statistical software (version 3.1.14), to explore the multivariate structure and the potential differences. The results were expressed as mean ± standard error, and the differences of all means were determined at *P* < 0.05. Furthermore, PCA and other cluster (dendrogram) analyses were carried out to understand the pattern of FA profiles variation among the crab populations.

## Results

### FA Profiles as Biomarkers for Crab Populations From Different Geographic Locations

#### Variation in FA Profiles Based on the Geographic Origin of Crab Populations

The FAs [saturated FAs (SFAs), monounsaturated FAs (MUFAs), PUFAs, and unsaturated FAs (UFA)] identified from the six Chinese mitten crab populations, and their total aggregated percentages are shown in Table 2. Generally, SFA abundance (C14:0, C16:0, C20:0, and C22:0) did not vary significantly between populations. However, crabs from H had significantly higher levels of margaric acid (C17:0), but lower levels of stearic acid (C18:0) than crabs from C, G, Gy, T, and Y. Pentadecylic acid (C15:0) is another SFA that showed significant differences (*P* < 0.05) between crab populations, and the highest level was found in crabs from H. Furthermore, pentadecylic acid levels were also higher in crabs from Y than in those from C. The percentage of aggregated SFA were significant (*P* < 0.05) and non-significant among the populations. While it was significantly higher in crabs from Gy than in those from G, T, and Y, there was no significant difference when comparing the Gy population with those from C and H. Similarly, there were no significant differences in SFA percentage when comparing crabs from G, T, and Y, with C and H.

**TABLE 2:** Fatty acid profiles of 6 Chinese mitten crab populations from different lakes.

	C	G	Gy	H	T	Y
C12:0	0.018 ± 0.002^a^	0.017 ± 0.003^a^	0.016 ± 0.003^ab^	0.017 ± 0.005^a^	0.012 ± 0.002^ab^	0.010 ± 0.003^b^
C14:0	0.418 ± 0.035	0.483 ± 0.017	0.411 ± 0.023	0.514 ± 0.029	0.460 ± 0.019	0.515 ± 0.036
C15:0	0.207 ± 0.011^c^	0.275 ± 0.009^bc^	0.235 ± 0.014^bc^	0.399 ± 0.033^a^	0.248 ± 0.009^bc^	0.285 ± 0.013^b^
C16:0	12.157 ± 0.237	12.434 ± 0.146	12.870 ± 0.199	12.076 ± 0.144	12.677 ± 0.236	12.222 ± 0.206
C16:1	4.381 ± 0.465^ab^	3.348 ± 0.114^b^	5.596 ± 0.692^a^	5.144 ± 0.676^ab^	3.509 ± 0.171^b^	3.250 ± 0.120^b^
C17:0	0.617 ± 0.032^b^	0.644 ± 0.028^b^	0.515 ± 0.042^b^	0.878 ± 0.092^a^	0.542 ± 0.025^b^	0.612 ± 0.022^b^
C18:0	7.707 ± 0.205^b^	7.368 ± 0.207^b^	6.988 ± 0.186^b^	6.080 ± 0.255^a^	7.328 ± 0.154^b^	7.328 ± 0.170^b^
C18:1	22.979 ± 0.747^b^	22.610 ± 0.295^b^	25.708 ± 0.765^a^	22.702 ± 0.962^b^	25.215 ± 0.375^ab^	23.026 ± 0.291^b^
C18:2	8.090 ± 0.771^a^	5.674 ± 0.343^ab^	4.590 ± 0.297^b^	5.183 ± 1.190^b^	5.736 ± 0.426^ab^	6.669 ± 0.433^ab^
C18:3	1.379 ± 0.085^ab^	1.225 ± 0.117^b^	2.245 ± 0.269^c^	2.463 ± 0.221^a^	0.847 ± 0.052^b^	0.956 ± 0.046^b^
C20:0	0.142 ± 0.014	0.174 ± 0.014	0.128 ± 0.010	0.118 ± 0.015	0.160 ± 0.042	0.197 ± 0.018
C20:1	0.768 ± 0.081^b^	0.871 ± 0.048^b^	0.790 ± 0.061^b^	1.089 ± 0.107^ab^	1.065 ± 0.046^ab^	1.367 ± 0.103^a^
C20:2	1.273 ± 0.129^bcd^	1.735 ± 0.053^ab^	1.040 ± 0.090^cd^	1.558 ± 0.105^abd^	1.215 ± 0.091^d^	1.866 ± 0.082^a^
C20:3	0.582 ± 0.058^ac^	0.407 ± 0.028^bc^	0.587 ± 0.026^ac^	0.486 ± 0.051^c^	0.291 ± 0.022^b^	0.353 ± 0.020^bc^
C20:4	6.337 ± 0.252^bc^	5.991 ± 0.221^bc^	6.882 ± 0.338^ac^	8.721 ± 0.894^a^	4.741 ± 0.366^b^	5.735 ± 0.218^bc^
C20:5	18.933 ± 0.650^ab^	20.281 ± 0.242^a^	17.495 ± 0.860^b^	17.933 ± 0.822^ab^	18.308 ± 0.261^ab^	18.731 ± 0.386^ab^
C22:0	0.109 ± 0.004	0.124 ± 0.003	0.112 ± 0.015	0.090 ± 0.006	0.116 ± 0.006	0.113 ± 0.010
SFA	25.754 ± 0.458^ab^	24.867 ± 0.306^b^	26.871 ± 1.258^a^	25.314 ± 0.462^ab^	25.051 ± 0.299^b^	24.532 ± 0.259^b^
MUFA	23.956 ± 0.764^bcd^	23.680 ± 0.264^b^	26.710 ± 0.725^ad^	24.025 ± 0.994^bc^	26.485 ± 0.422^d^	24.659 ± 0.272^abcd^
PUFA	50.289 ± 1.175^a^	51.453 ± 0.387^a^	46.419 ± 1.168^b^	50.660 ± 1.010^a^	48.463 ± 0.610^ab^	50.809 ± 0.444^a^
UFA	74.245 ± 0.458^ab^	75.132 ± 0.306^a^	73.129 ± 0.532^b^	74.686 ± 0.463^ab^	74.949 ± 0.299^a^	75.468 ± 0.259^a^

*Values are means ± SE of 8 crabs from each lake. Means with at least one same superscript in the same row are not significantly different (p < 0.05).*

The percentage of aggregated MUFAs was significantly higher (*P* < 0.05) in crabs from T than in those from G. However, the MUFA percentage in crabs from T was not significantly different from those sampled from C, Gy, and Y. Furthermore, no differences were observed when comparing crabs from G with those from C, H, and Y. The percentage of PUFAs was significantly higher in crabs from C, G, H, and Y than in those from Gy, however, no significant differences were observed when crabs from T were compared to those from Gy, and when compared to those from C, G, H, and Y. The aggregated percentage of UFAs (MUFAs and PUFAs) was found to be higher in crabs from G, Y, and T than in those from Gy (*P* < 0.05), but there was no difference between Gy, C, and H populations. The percentage of aggregated UFAs was also comparable between C, G, Y, and H populations.

#### Dendrogram Showing the Relationship Between Crab FA Composition and Geographic Location

The FA profiles of the six crab populations varied significantly according to their geographic origins. This suggests that these FA profiles may be dependent on the geographical distribution of these crabs (Figure 1A). To verify this hypothesis, we created a dendrogram using the FA profile dataset (Figure 1B), which divided the six populations into two groups: Y, G, and T clustered into the first clad, and H, Gy, and C clustered into another clad. The resulting cluster style conformed to the geographical distribution of the crab populations. Similar patterns were shown by principal component analysis (PCA) (Figure 1C). On the dendrogram, the lakes Y and T belonged to geographical branch A, and H and Gy belonged to geographical branch B. G and C belonged to geographic branches that connect A and B branches (Figure 1B).

### Read Sequencing, Assembly, Mapping, and Characterization

The total numbers of raw sequence reads for the intestinal contents and mucosa samples were 4,218,454 raw reads (with an average of 95,874 reads per sample) and 3,947,857 total clean reads (with an average of 89,724 reads per sample) respectively (Supplementary Table S1). The clean reads were deposited into the Sequence Read Archive database at NCBI (SRR12277956-SRR12277999).

### Gut Bacterial Community Structure Varied According to the Geographic Origin of the Crab Population

#### Alpha and Beta Diversity

The alpha diversity parameters (Shannon, Simpson, ACE, and Chao1) were normalized and calculated for all crab populations at a 97% identity threshold (Supplementary Table S2). The alpha diversity parameters plotted (Supplementary Figures S1A–D, S3B). The results showed that crabs from T had the lowest bacterial species richness and alpha diversity. Those from H had the highest bacterial species richness, while those from Y had the highest diversity. Furthermore, crabs from H had the highest weighted and unweighted species diversity (Supplementary Figures S1C,D). Crabs from G had the lowest weighted species diversity, and those from T had the lowest unweighted beta diversity. Results from the rarefaction and rank abundance curves indicate that the number of samples taken are reasonable (Supplementary Figure S2).

Rarefaction and rank abundance curves are common curves that describe the diversity of samples in a group. The Rarefaction curve clearly shows how reasonable the amount of sequencing data is and indirectly reflects the richness of the sample species. When the curve is flat, it indicates that the sequencing data volume is reasonable, and more data volume will only produce a small number of new species (OTUs). To plot the rarefaction, and rank abundance curves, a certain amount of sequencing data were randomly selected from the sample, and the number of species represented by this data (i.e., the number of OTUs) was counted.

The rank abundance curve shows the relative species abundances (OTU numbers) starting from the most abundant to the least. To obtain the corresponding order number, find the OTU sort code on the abscissa and then the corresponding relative abundance of OTUs (or the species abundance for each sequence) on the ordinate, and connect these points with broken lines. Normally, a rank abundance curve reflects the richness and evenness of species in the sample. The species richness is reflected by the width of the curve across the abscissa. The greater the species richness, the wider the area of the curve across the horizontal axis. In the vertical direction, the smoothness of the curve reflects the evenness of species in the sample. The flatter the curve, the more homogeneous the species distribution (Lundberg et al., 2013).

Our rarefaction curves tended to be flat, indicating that the number of samples sequenced were reasonable and could reflect the real situation of the samples tested (Supplementary Figure S2A). The abundance indices for the 6 populations were compared, which first decreased and then increased in crabs from H and Y, with the highest in crabs from H and the lowest in crabs from T (Supplementary Figure S2B). Looking along the abscissa, at the width of the rank abundance curve, the crabs from H had the widest curve and the highest species richness, whereas crabs from T had the narrowest curve and the lowest species richness, which was consistent with the results from the dilution curve. The vertical axis shows that the crabs from H had the smoothest curve, whereas, those from Y had the steepest curve. Therefore, in terms of species uniformity, crabs from H showed the greatest uniformity, and those from Y were the least uniformed.

#### OTU Structure at Different Classification Levels

As described in section “FA Profiles as Biomarkers for Crab Populations From Different Geographic Locations,” the FA profiles of the six crab populations can be used to distinguish geographic origin. The differences observed between the FA profiles of different crab populations have been attributed to variations in gut bacteria, which are influenced by different environmental conditions prevalent at each geographical location. Hence, in this study, we analyzed the type and abundance of intestinal microbial communities found in the six crab populations. The analysis revealed that a total of 1066 different bacterial species were shared among the six crab populations, and crabs from T had the lowest number of bacterial species (87) (Supplementary Figure S3A).

A species classification tree was constructed (from the top 10 relative abundance at the genus level, by default) (Pond et al., 2006), and the kingdom, phylum, class, order, family, genus, and species were arranged from left to right (Supplementary Figure S4). It showed the OTU structure of all six crab populations at different taxonomic levels. At the phylum level, the most dominant bacteria in all crab populations were Tenericutes. However, there were no significant differences in the proportions of Tenericutes among these populations. Crabs from Gy had the highest proportions of Bacteroidetes at all levels below and inclusive of the phylum (class, family, and genus). Crabs from C had the highest number of Firmicutes, followed by crabs from Y and then the other populations. Crabs from G had the highest number of Proteobacteria. At the genus level, the distribution for specific bacterial populations was similar to that of the phylum. Among the identified bacteria, *Shewanella* was only found in crabs from G, Y, and Gy based on the 0.01 level (Supplementary Figure S4 and Supplementary Table S3).

**FIGURE 2:**
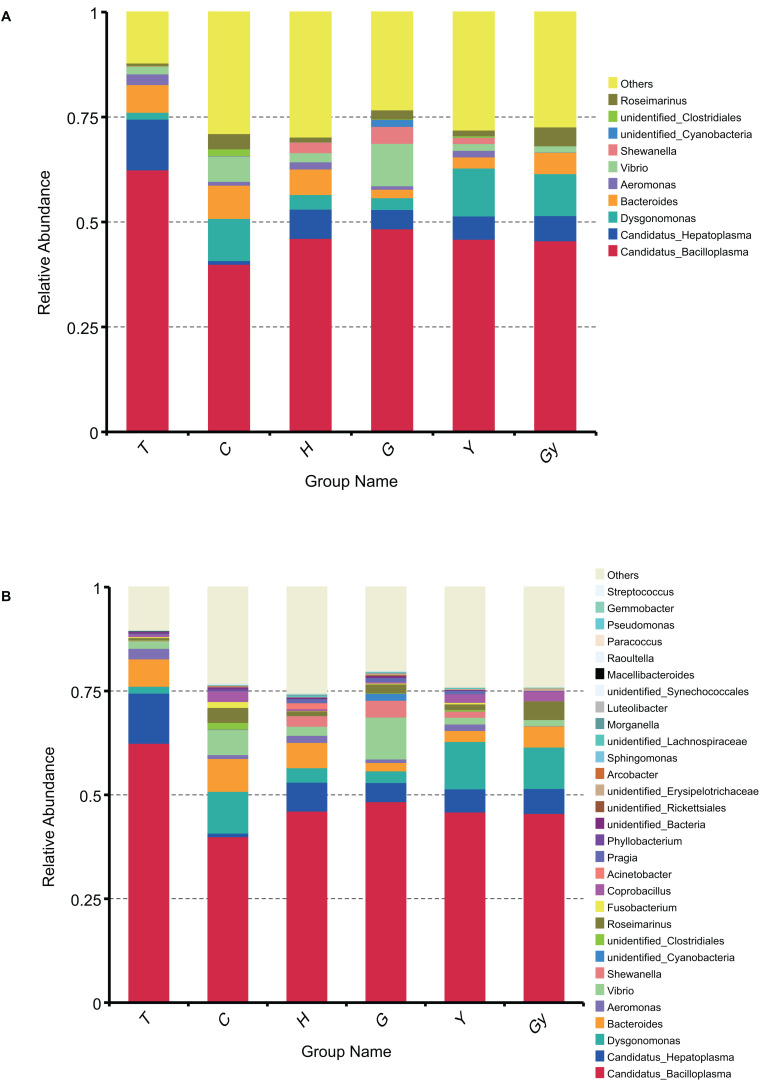
Bar chart of gut bacteria relative abundance on genus level. **(A)** Relative abundance of top 10 genera. **(B)** Relative abundance of top 30 genera.

To determine the dominant genus structure in each group, the top 10 and 30 genera based on relative abundance were selected to generate a cumulative histogram (Figure 2). *Aeromonas*, *Bacteroides*, *Dysgonomonas*, *Candidatus Hepatoplasma*, and *Candidatus Bacilloplasma* were the dominant genera (Figure 2A). Among these, *Candidatus Bacilloplasma* was the most prevalent, present in more than 0.5% of crabs from T. The top 10 genera accounted for 75% of nearly every population. This distribution did not significantly change when a further 20 genera were added to the dataset (Figure 2B). To display the phylogenetic relationship of the bacterial species found in the crab populations, the top 100 genera obtained from multiple sequence alignment and analysis of relative abundance were used to construct a LEfSe Cladogram (Supplementary Figure S5A).

The relative abundance of each bacterial genus in each crab population is reflected by its proportional representation on the outer ring (Supplementary Figure S5A). Tenericutes were the most abundant, Bacteroidetes, the third-most abundant, and Proteobacteria were the least abundant among the crab populations. To identify the genera that were clustered in specific samples, a heatmap (a heat map chart) was created from the top 35 genera, based on their abundance and taxonomic annotation (Supplementary Figure S5B).

### The Evolutionary Distance and Relationship Between the Bacterial Communities Found in the Different Crab Populations

#### Genetic Differences Among the Bacterial Communities

**FIGURE 3:**
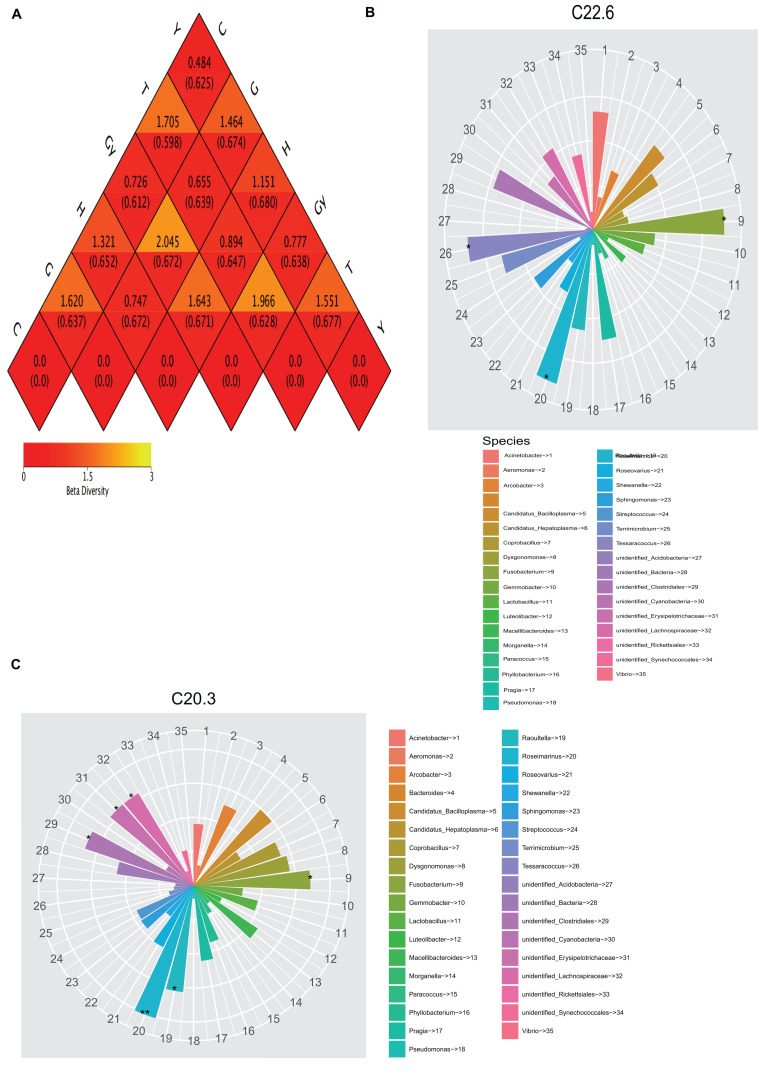
Heatmap of beta diversity and relationship between fatty acid profile and bacterial community. **(A)** Weighted and unweighted Unifrac distances. **(B)** Relationship between C22:6 fatty acids and gut bacterial community. **(C)** Relationship 20:3 fatty acids and gut bacterial community.

Both weighted and unweighted Unifrac distances were used to calculate the dissimilarity coefficient between the two populations. The smaller the value, the smaller the difference in diversity between these two populations. A heatmap based on weighted and unweighted Unifrac distances was created (Figure 3A). For weighted Unifrac distance, the dissimilarity coefficient between groups Y and C was 0.484, which was the smallest difference among the six populations, indicating that this pair had the greatest similarity in species diversity. The largest dissimilarity coefficient (2.045) was observed between crabs from G and Gy, indicating that these two populations differed the most in terms of species diversity. For the unweighted Unifrac distance, the dissimilarity coefficient for crabs from C and T was 0.277, which was the smallest value, whereas the highest dissimilarity coefficient (0.680) was between crabs from Y and H.

#### Biomakers for Different Populations as Explored by LefSe

Having established the dissimilarity coefficients among the six crab populations, specific bacteria and their evolutionary relationships were subsequently analyzed (Supplementary Figure S6A). *Prolixibacteraceae*, *Lachnospiraceae*, and *Erysipelotrichaceae* were found in crabs from Gy only, whereas unidentified Bacteroidales were only found in crabs from Y. At the order level, Bacteroidiales and Clostridiales were only found in populations from C. At the phylum level, Tenericutes and Proteobacteria were observed in crabs from T and G; *Alphaproteobacteria* were only found in crabs from H. The relative abundance of each bacterial genus in the six populations is shown in Supplementary Figure S6B.

#### The Bacterial Community Structure in Different Crab Populations and Their Specific Taxa

**FIGURE 4:**
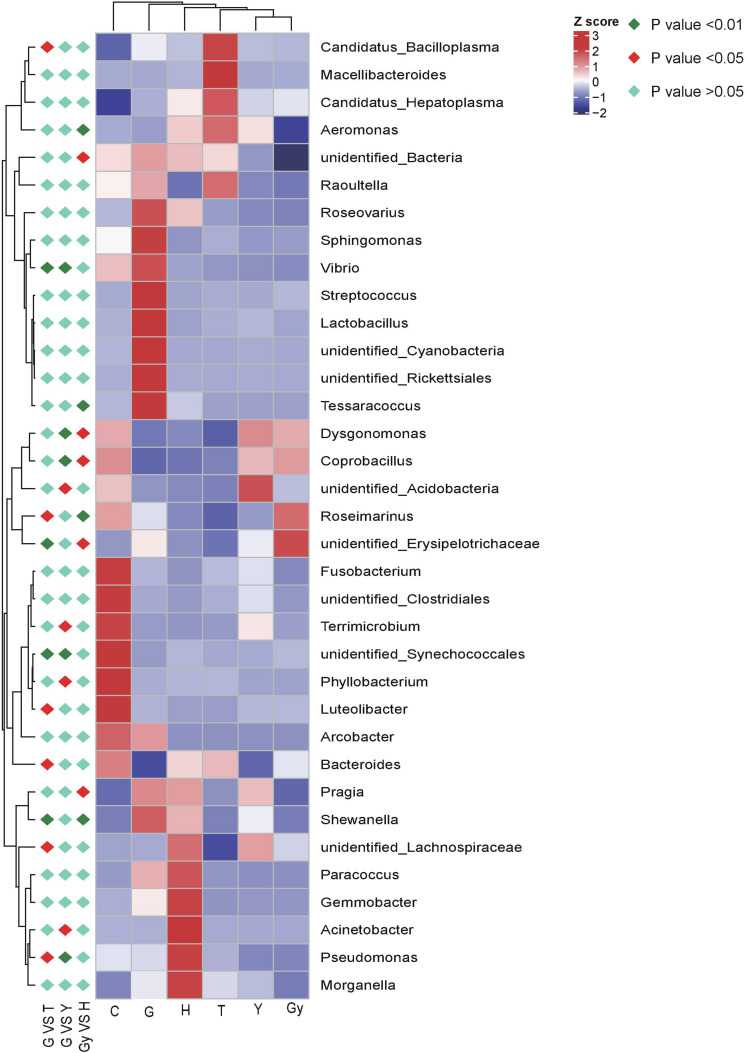
Heat map of the correlation matrix between crab population and the bacteria communities. Red orange tones indicate a positive correlation between crab population and the bacteria communities; blue and yellow white indicate absence of correlation as for example, pragia has no correlation with crabs from Gy, meaning pragia was not found in crabs from Gy.

The metaStat method was used to identify bacteria that were significantly different among the six crab populations. The differences in the abundance of bacterial species distributed among the populations were presented, including the 12 bacterial phyla that showed the most significant differences and those that did not show a difference. All bacterial genera structures that showed significant abundance by pairwise comparison (*n* = 5 per crab population) were plotted (Figure 4). Using the metaStat test, 35 genera were identified from 3 crab population pairs (Gy-H, G-Y, and G-T).

#### Evolutionary Analyses of the Bacterial Community Structures Found in Crab Populations Using PCA

**FIGURE 5:**
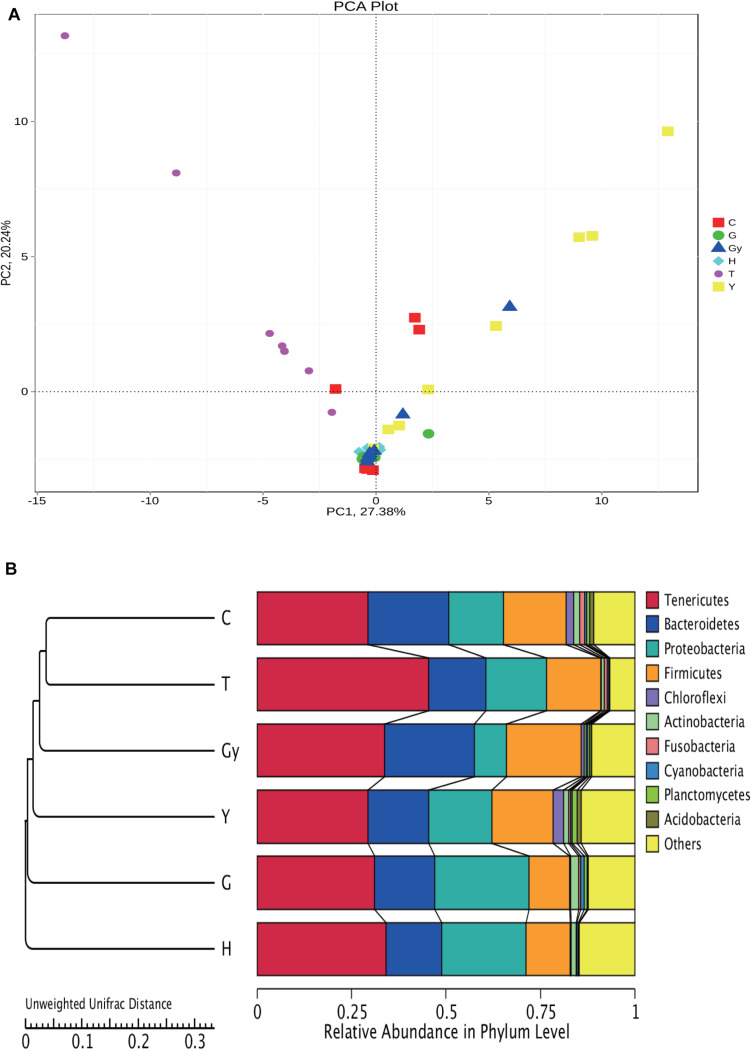
Cluster analysis of all groups based on bacterial community. **(A)** PCA plot is illustrating differences in gut bacterial communities of different crab population. **(B)** UPGMA clustering tree based on weighted Unifrac distance.

In the present study, we carried out a PCA of the bacterial genera found in microbial communities from the six crab populations (Figure 5A). PC1 divided the samples into two groups: one group was composed of crabs from Y, C, and Gy, and the other group consisted of crabs from T, H, and G. PC2 also divided the crab populations into two groups. One group consisted of crabs from T and Y, and a small number of those from Gy and C, while crabs from H and G were segregated to the other group. PC1 and PC2 mainly divided the six crab populations into two groups, which indicate that there were significant differences in bacterial composition among the crab populations.

To study the similarity between the crab populations, the unweighted pair group method with arithmetic mean (UPGMA) method was used. This clustering analysis is based on the unweighted Unifrac distance matrix (Figure 5B). The left side is the UPGMA clustering tree, and the right side is the relative abundance of the bacterial communities at the phylum level. Furthermore, it can be s the distance between the C and T groups was the smallest, and they were then grouped with Gy, Y, G, and H. Among these populations, H and C were the furthest apart.

### Relationship Between FA Profiles and Bacterial Community Structures

To determine the relationship between crab FA profiles and their gut microbial community compositions, we compared two dendrograms that depict FA profiles and bacterial community structures. The results showed that the positions of lakes of crab origin were the same on the two dendrograms (FA profiles and bacterial community structures dendrograms), except for H and Y that swapped positions on these dendrograms (Figures 1B, 2B). To determine the cause of this difference, the relationship between FA and bacterial community structure was investigated. A mantel test was carried out comparing each FA that showed a significant difference among the crab populations with the 16S sequence OTU dataset (Supplementary Table S4). To determine the relationship between FA profiles and bacterial community structure, the analysis was performed in two phases. In the first phase, all FA profiles for each crab population were correlated with the bacterial OTU dataset, which showed that FAs were significantly related to the bacterial community. In the second phase, all FA profiles, which were significantly different among the six crab populations, were correlated with the bacterial OTU dataset. This showed that significant variation in the relative abundance of specific FA could be caused by different bacterial communities. Canonical correspondence analysis (CCA) and distance-based redundancy analysis (dbRDA) were also used to further understand how FA composition is related to the bacterial communities (Supplementary Tables S5, S6). All FAs that were significantly different were identified, and the FAs podocarpic acid (C20:3) and cervonic acid (C22:6) that varied in concordance with changes in the bacterial genera structures were analyzed by Spearman’s correlation (*P* < 0.01) and they were plotted on the cladograms (Figures 3B,C). The genera that correlated with podocarpic acid (C20:3) and cervonic acid (C22:6) changes were *Fusobacterium* and *Roseimarinus*. The results from Spearman’s correlation analysis of FA composition and bacterial community structure at the genus level are shown in Supplementary Table S7.

## Discussion

### FA Profiles as Biomarkers for the Geographic Location of Crab Populations

Our study showed that the FA profiles of the six crab populations varied significantly and were dependent on the geographic origin of these crabs (Table 2 and Figures 1A,B, 2). Similarly, Sillero-Ríos et al. (2018) reported significant variations in the FA composition of *Octopus vulgaris* inhabiting three coastal areas in the West Mediterranean Sea. Significant differences in 22:6 n-3 (DHA, docosahexaenoic acid) and 22:5 n-3 (EPA, eicosapentaenoic acid) FA composition was found in the mantles of the *O. vulgaris* sampled from these areas. Similarly, Rude et al. (2016) demonstrated that the FA profiles of the bluegill differed significantly on the basis of habitat, suggesting that FA profiles can be used as biomarkers for fish habitat. Other studies conducted on terrestrial animals have also shown that the FA composition of milk, muscle, and other tissues can be linked to the geographic origin of these animals (Rutkowska et al., 2015; Kumar et al., 2016; Barłowska et al., 2018). Furthermore, in a study that focused on microbiota and lipids, it was found that the composition of lipids (in particular that of PUFAs) and microbiota in milk differed in animals raised in different geographic locations (Kumar et al., 2016). One of the reasons for this variation is the environment. In a study directed to see how goat milk is influenced by the environment, it was found that raising goats in certain geographic regions increased the proportion of beneficial FAs in milk and enhanced the qualities valued by consumers (Barłowska et al., 2018). In addition, in cow milk it was discovered that the high levels of linoleic acid (CLA), vaccenic acid (VA), total C18:1trans, and gamma-linolenic acid (GLA, C18:39c12c15c n-6), alpha-linolenic acid (ALA, C18:39c12c15c), were indicative of the Polish mountainous regions while the short-chain saturated FAs (SCFA, C4:0–C11:0), of the lowland (Rutkowska et al., 2015). Therefore, our results suggest that the FA profiles can be used as biomarkers for the geographic origin of crabs.

### FA Composition Is Influenced by the Bacterial Community Structure in Crab Guts

Numerous studies have reported that intestinal microbial community structure influences FA composition. For example, using a comprehensive multi-omics approach, gut microbiota has been shown to induce MUFA generation by stearoyl-CoA desaturase 1 and PUFA elongation by FA elongase 5, leading to significant changes in the glycerophospholipid acyl-chain profiles (Kindt et al., 2018). Similarly, in the present study, our analyses generated patterns that linked FA profiles with gut bacterial communities (Figures 1B, 2B and Supplementary Table S4). Similar variations were observed for both FA compositions and gut bacterial communities based on the geographic origin of the crab populations. However, consistent with other associative studies of the gut microbiota, the major challenge is to explain the underlying biological mechanisms and to establish the cause of the observed relationships. Furthermore, information on the relationship between gut bacterial communities and FA profiles in crustaceans is generally lacking. Nevertheless, studies conducted on humans, rodents (mice), zebrafish, and other animals provide possible biological mechanisms that could explain the relationship between the bacterial community and FA composition observed in the present study (Ley et al., 2006; Semova et al., 2012; Chakraborti, 2015). Ley et al. (2005) reported significant differences in the proportions of Firmicutes and Bacteroidetes in mice. In obese mice, the relative proportions of Firmicutes and Bacteroidetes significantly increased and decreased, respectively, in comparison to normal mice. Similarly, obese children had a higher Firmicutes to Bacteroidetes ratio than lean children (Bervoets et al., 2013). The increase in the abundance of Firmicutes has often been linked with an increase in lipid storage due to obesity (Ley et al., 2005, 2006). Other gut bacteria linked to FA metabolism, energy intake, and storage include saccharolytic bacteria, lactobacilli, and staphylococci (Ley et al., 2006; Pachikian et al., 2011; Chakraborti, 2015). One study proposed that under certain conditions in humans and mice, some saccharolytic bacteria species are involved in fat storage and metabolism (Ley et al., 2008). Some *Staphylococcus* species have been positively linked with energy intake in children (Bervoets et al., 2013). Moreover, Million et al. (2012) reported that *Lactobacillus* species, via its role in FA metabolism, are linked to body weight gain and obesity in adults. Additionally, many studies have shown that Firmicutes, in comparison to other bacterial phyla, have a strong association with FA absorption, metabolism, and storage (Ley et al., 2006; Semova et al., 2012; Chakraborti, 2015). In zebrafish, an increase in the abundance of Firmicutes was correlated with an increase in the number of lipid droplets and the promotion of FA absorption (Semova et al., 2012). In the present study, significant differences in the proportions of Firmicutes and Bacteroidetes were observed (Supplementary Figure S4 and Supplementary Table S3). Thus, the variations in gut bacterial communities, especially in the relative abundance of Firmicutes and Bacteroidetes, may be responsible for the variations in the FA composition of the different crab populations.

Furthermore, other studies attempted to verify and explain the relationship between gut bacterial communities and FA profiles. For example, Kindt et al. (2018) used short-term antibiotic treatment to manipulate the gut microbial ecosystem of specific pathogen-free (SPF) animals (mice) and found that the relative FA 16:0, FA 20:3, FA 20:4, and FA 22:6 levels were affected by this treatment. Similarly, in laying Japanese quails (*Coturnix coturnix japonica*), the FA composition of liver lipids can be modified by manipulating gut microflora (Furuse et al., 1992). In the present study, the presence of podocarpic (C20:3) and cervonic (C22:6) acids were explored by CCA and dbRDA overlapping analysis (Figures 2, 3A and Supplementary Tables S5, S6). We found that changes to the abundance of some *Fusobacterium* and *Roseimarinus* bacteria were correlated with alterations in the composition of FA 20:3 and FA 22:6 in the crabs. Similarly, in Gy and C crab populations, FA 20:3 content was higher than that of other populations, and this correlated with higher Bacteroidetes and Firmicutes abundance (Figures 4B,C). The bacteria were identified by Spearman’s correlation analysis to determine the influence of FA composition (Supplementary Table S6).

### Gut Bacterial Community Structure Is Associated With Geographic Location

Generally, the bacterial community structure in the intestinal tracts of fishes from different lakes is different, which may be dependent on many factors (such as food source, water quality, and lifestyle) in the lake. For example, in a study of cichlids living in two crater lakes, the gut microbiota composition, in particular Oceanospirillales (52.28%; halotolerant or halophilic bacteria), differed between the two populations (Franchini et al., 2014). However, both the developmental stage and the geographical location are important determinants of gut bacterial composition in mosquitoes (Bascuñán et al., 2018). Thus, both the developmental stage of an animal and its geographical location determines its gut bacterial composition (Finkel et al., 2011). Variations in microorganisms associated with a host organism were also verified in trees of the same species that were grown in different climate regions. The trees hosted distinct microbial rhizosphere and phyllosphere communities (Finkel et al., 2011). The abovementioned findings are consistent with the findings of our study. In the present study, we found significant variations in gut bacteria at the phylum and lower taxonomical levels according to the geographical origin of the crab population (Figures 2, 3A, 5 and Supplementary Table S6). However, geographic location did not have a significant effect on gut microbial communities of farmed sea bream (*Sparus aurata*) and sea bass (*Dicentrarchus labrax*) (Bass et al., 2018).

Environmental characteristics are among the factors that determine bacterial community composition. A mouse study showed that the type of water consumed significantly influenced the composition of gut microbiota (Dias et al., 2018). In fish, the gut microbial community structure can be affected by water and other substances found in the water. The addition of microalgae to rearing tanks affected the composition and dynamics of the microbial communities in the rearing water (Giménez Papiol et al., 2018). Total organic carbon, n-alkanes, petroleum hydrocarbons, and other compounds were found to be highly related to the bacterial community composition at different depths of water and sediments, originating from various regions of the East Mediterranean Sea (Polymenakou et al., 2005). Food choices also play an important role in shaping the composition and activity of gut microbiota (Zhao et al., 2018). Another factor that played a role in the geographic determination of bacterial composition is ecology. In a study by Sullam et al. (2012), the composition of fish gut bacteria was found to be shaped by host phylogeny, the water salinity, and the trophic level. In lakes, the most important source of bacteria is the resident community, however, dispersal of these sources have limited immediate effects on the gut bacterial community (Comte et al., 2017).

Fish gut is in a continuous contact with the surrounding water, however, it has been shown that many microbial taxa present in surrounding waters are not found in the gut of fish and vice versa. This suggests that host associated factors strongly influence the gut bacteria community composition compared to environmental factors (Schmidt et al., 2015). In the present only gut bacteria communities were evaluated, the environment parameters and the microbial communities in the surrounding water were not evaluated. This is a major limitation of our study, hence, it was difficult to quantify and identify the exact factors responsible for the observed variations in gut bacteria communities. Nonetheless, the standard water parameters for the lakes where the crab populations were sampled can be found at (GB3838-2002).

While some recent studies suggest both the host associated and environmental factors to be significantly contributing to the fish gut microbial community structure (Dehler et al., 2017), others suggest a stronger influence of the host selective pressures (host genetics) (Li et al., 2017). Therefore, gut microbiota assembly in fish may be primarily controlled by deterministic processes due to host-dependent restrictions (Yan et al., 2016). Individuals of the same fish species occupying different habitats often host variable amount and type of gut microbial community that tend to correspond with variation in their genetic constitution (Dolan, 2005; Allan and Max, 2010; Neuman et al., 2016). The genetic background of the crab populations included in the present was evaluated in our other work (Munganga et al., unpublished), the analysis showed no significant genetic differentiation among the six populations, no geographical background clustering of the populations was observed.

The exact degree with which each of these factors influence the gut microbiome is not known. Clearly, it is difficult to distinguish the host specific and environmental effects on fish gut microbiota. The difference in feeding behavior of different species adds to the problem of investigating the role of each of these factors. It will be essential in future studies to include additional factors such as trophic levels, potential influences of environmental parameters (e.g., diet composition, food chain dynamics, water depth and temperature, geographic location), and compare variations according to season.

## Conclusion

Similar to mammals, fish gut microbial communities play an integral part in host intestinal digestion, metabolic homeostasis, physiology, immune response, and response to disease treatment. Therefore, understanding the principles governing gut microbial assemblage and maintenance within the intestine of animals and how they interact with the host and the environment has become a primary focus. In concordance with results from previous studies, the current study presents interesting results on the interaction between the gut bacterial community, host organism, and the environment. The FA profiles of six Chinese mitten crab populations varied significantly according to geographical origin. Similar variations were also observed in the composition of the gut, which varied according to geographical origin. Further analysis of FA profiles and gut microbial community generated patterns that linked the two parameters. The FA profiles were found to be significantly related to the composition of the bacterial community in crabs from each geographic area. Hence, it was observed that the gut microbial community seems to contribute significantly to the FA composition of the Chinese mitten crab. This provides new insights that could contribute to future research on the nutrition of Chinese mitten crabs and other related areas. Furthermore, variations in FA composition were also shown to be a potential biomarker for crab geographical background. Our study is only an early analysis of the relationships between FA composition and gut bacteria community in the Chinese mitten crab, and further studies are needed to provide a deeper understanding of these interactions. These studies will help us unravel the complexity of host-gut microbial interactions for possible applications in aquaculture and species conservation.

## Data Availability Statement

The sequencing data has been deposited into a publicly accessible NCBI repository (accession: PRJNA646327).

## Ethics Statement

The animal study was reviewed and approved by the Wuxi Municipal Bureau on Science and Technology.

## Author Contributions

SS, YT, and PX conceived the study and contributed to the design of the experiments. BM, FD, JY, JL, FY, MW, XH, XL, and RB performed all the experiments. All authors contributed to the drafting of the manuscript.

## Conflict of Interest

The authors declare that the research was conducted in the absence of any commercial or financial relationships that could be construed as a potential conflict of interest.

## Footnotes

1 http://ccb.jhu.edu/software/FLASH/

2 http://qiime.org/index.html

3 http://www.drive5.com/usearch/manual/uchime_algo.html

4 http://drive5.com/uparse/

5 http://greengenes.lbl.gov/cgi-bin/nph-index.cgi

6 http://sourceforge.net/projects/rdp-classifier/

7 http://www.drive5.com/muscle/

8 http://www.r-project.org/

## Supplementary Material

Supplementary Figure 1 Boxplot of Alpha-diversity indices. Alpha diversity indexes are multiple indexes reflecting consistency and abundance. **(A)** Ace and **(B)** Shannon, **(C)** Simpson, and **(D)** Chao1 indices reflect the OTU abundance in samples. Boxes represent the interquartile range between the first and third quartiles (25th and 75th percentiles, respectively) and inside the box the horizontal line shows the median.

Supplementary Figure 2 Dilution and rank abundance curves for all gut bacteria community. **(A)** Rarefaction curves and **(B)** Ranks abundance curves; the x-axis shows the bacterial species and the y-axis shows the relative abundance of the bacteria. Each curve in the graph represents a different crabs and different colors are used to show their lake of origin or group they belong to. As the sequencing depth increased, the number of gut bacteria increased the relative abundance increased in rarefaction curves and decrease in rank abundance curves.

Supplementary Figure 3 Core bacterial number among all groups and Shannon index box of six crab populations. **(A)** Venn diagram showing gut bacteria in each crab population. **(B)** Box plot of Shannon index showing the differences in the gut bacterial community of the six crab populations.

Supplementary Figure 4 Gut bacteria species-specific dendrogram of six crab population based on their geographical origin. The pie chart with different colors in the circle consist of crabs from different lakes. The size of each color in the pie is the proportion of the relative abundance of bacteria community from the corresponding lake. The numbers below the taxonomy name represents the average relative abundance percentage of all groups in this level (there are two numbers).

Supplementary Figure 5 Cladogram of LEfSe and Heat map of the correlation between gut bacteria and crab’s geographical origin. **(A)** Cladogram of LEfSe analysis showing abundance of microbial taxa in the six crab populations. The inner circle is the phylogenetic tree constructed by the representative sequences of genera. The color of branches represents their corresponding phylum, each color represents a phylum, and the outer circle represents the relative abundance of each genus in each group. **(B)** Heat map of the correlation matrix between crab population and the bacteria communities at phylum level. The horizontal axis are the different bacteria phyla represented by different color and on the horizontal axis are the lakes of crab origin. Red orange tones indicate a positive correlation between crab population and the bacteria communities; blue and yellow white indicate absence of correlation as for example.

Supplementary Figure 6 LefSe analysis. **(A)** The cladogram diagram shows the gut bacteria community species with significant differences in the six crab populations at phylum to genus level. The different colors represent different lakes and their corresponding bacteria. **(B)** Species with significant difference among the different crab populations have LDA score greater than the estimated value; the default score is 4.0. The length of the histogram is equivalent to the LDA score.

Supplementary Table 1 Read sequencing, assembly, mapping, and characterization.

Supplementary Table 2 Alpha and beta diversity parameters.

Supplementary Table 3 Out table and relative abundance.

Supplementary Table 4 Mantel test between significant fatty acid profile difference among all populations and 16S sequence OTU data set.

Supplementary Table 5 CCA for effective environment factor capturing.

Supplementary Table 6 dbRDA for effective environment factor capturing.

Supplementary Table 7 Spearman correlation analysis between fatty acid profile and each bacterial on genus level.
