# Supplementary material for: Relationship Between the Fatty Acid Profiles and Gut Bacterial Communities of the Chinese Mitten Crab (Eriocheir sinensis) From Ecologically Different Habitats
Source: Front Microbiol. 2020 Oct 15;11:565267. doi: 10.3389/fmicb.2020.565267 (PMC7593381; doi:10.3389/fmicb.2020.565267)
Supplement: Supplementary Figure 1 — Boxplot of Alpha-diversity indices. Alpha diversity indexes are multiple indexes reflecting consistency and abundance. (A) Ace and (B) Shannon, (C) Simpson, and (D) Chao1 indices reflect the OTU abundance in samples. Boxes represent the interquartile range between the first and third quartiles (25th and 75th percentiles, respectively) and inside the box the horizontal line shows the median. [file Image_1.pdf]

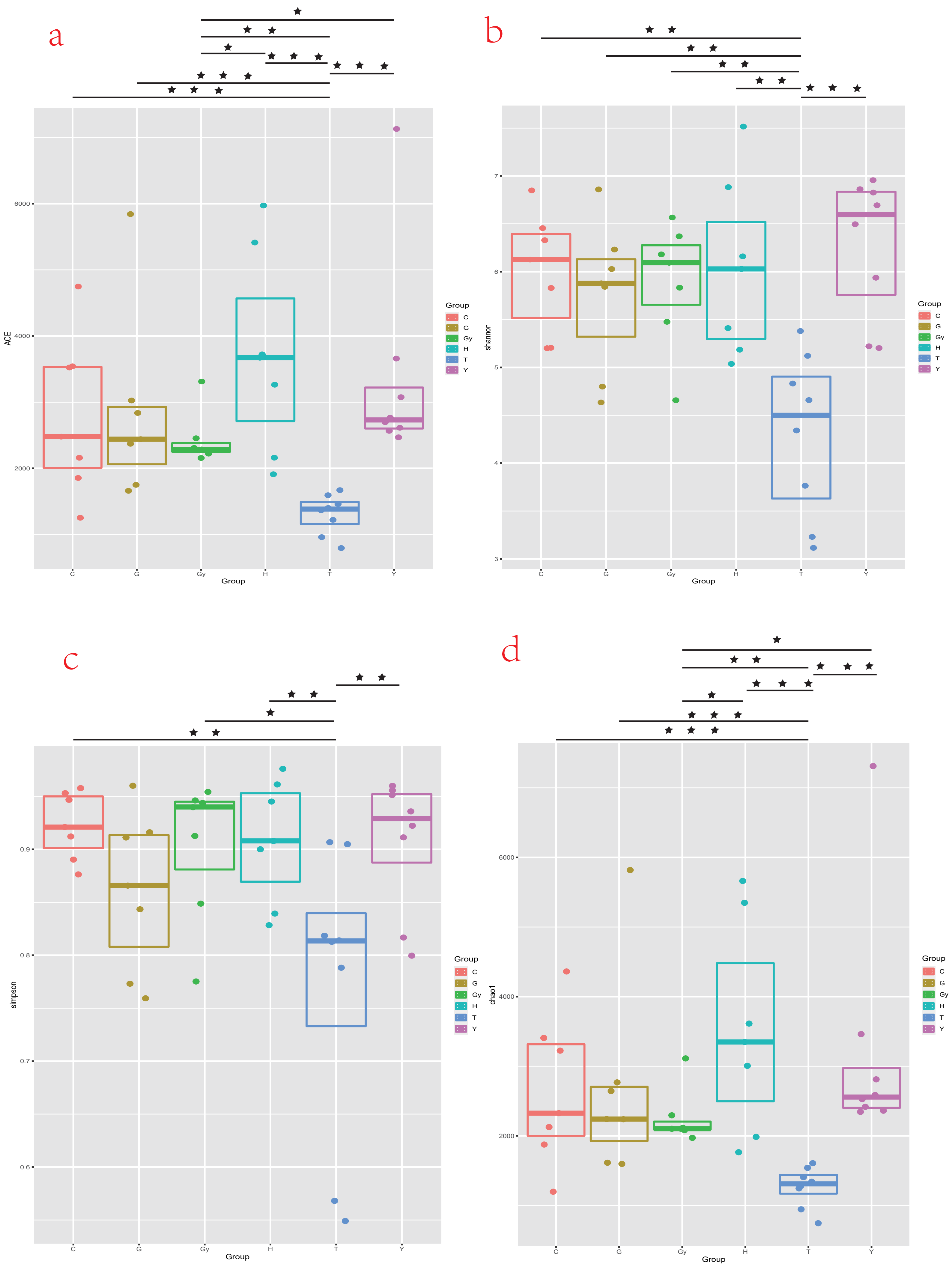
a
ACE
6000
4000
2000
C
G
Gy
H
T
Y
Group
Group
C
G
Gy
H
T
Y
b
shannon
7
6
5
4
3
C
G
Gy
H
T
Y
Group
Group
C
G
Gy
H
T
Y
c
simpson
0.9
0.8
0.7
0.6
C
G
Gy
H
T
Y
Group
Group
C
G
Gy
H
T
Y
d
chao1
6000
4000
2000
C
G
Gy
H
T
Y
Group
Group
C
G
Gy
H
T
Y

Supplementary Fig. S1 Boxplot of Alpha-diversity indices. Alpha diversity indexes are multiple indexes reflecting consistency and abundance. **(A)** Ace and **(B)** Shannon, **(C)** Simpson, and **(D)** Chao1 indices reflect the OTU abundance in samples. Boxes represent the interquartile range between the first and third quartiles (25th and 75th percentiles, respectively) and inside the box the horizontal line shows the median.
