# Supplementary material for: Relationship Between the Fatty Acid Profiles and Gut Bacterial Communities of the Chinese Mitten Crab (Eriocheir sinensis) From Ecologically Different Habitats
Source: Front Microbiol. 2020 Oct 15;11:565267. doi: 10.3389/fmicb.2020.565267 (PMC7593381; doi:10.3389/fmicb.2020.565267)
Supplement: Supplementary Figure 2 — Dilution and rank abundance curves for all gut bacteria community. (A) Rarefaction curves and (B) Ranks abundance curves; the x-axis shows the bacterial species and the y-axis shows the relative abundance of the bacteria. Each curve in the graph represents a different crabs and different colors are used to show their lake of origin or group they belong to. As the sequencing depth increased, the number of gut bacteria increased the relative abundance increased in rarefaction curves and decrease in rank abundance curves. [file Image_2.pdf]

a

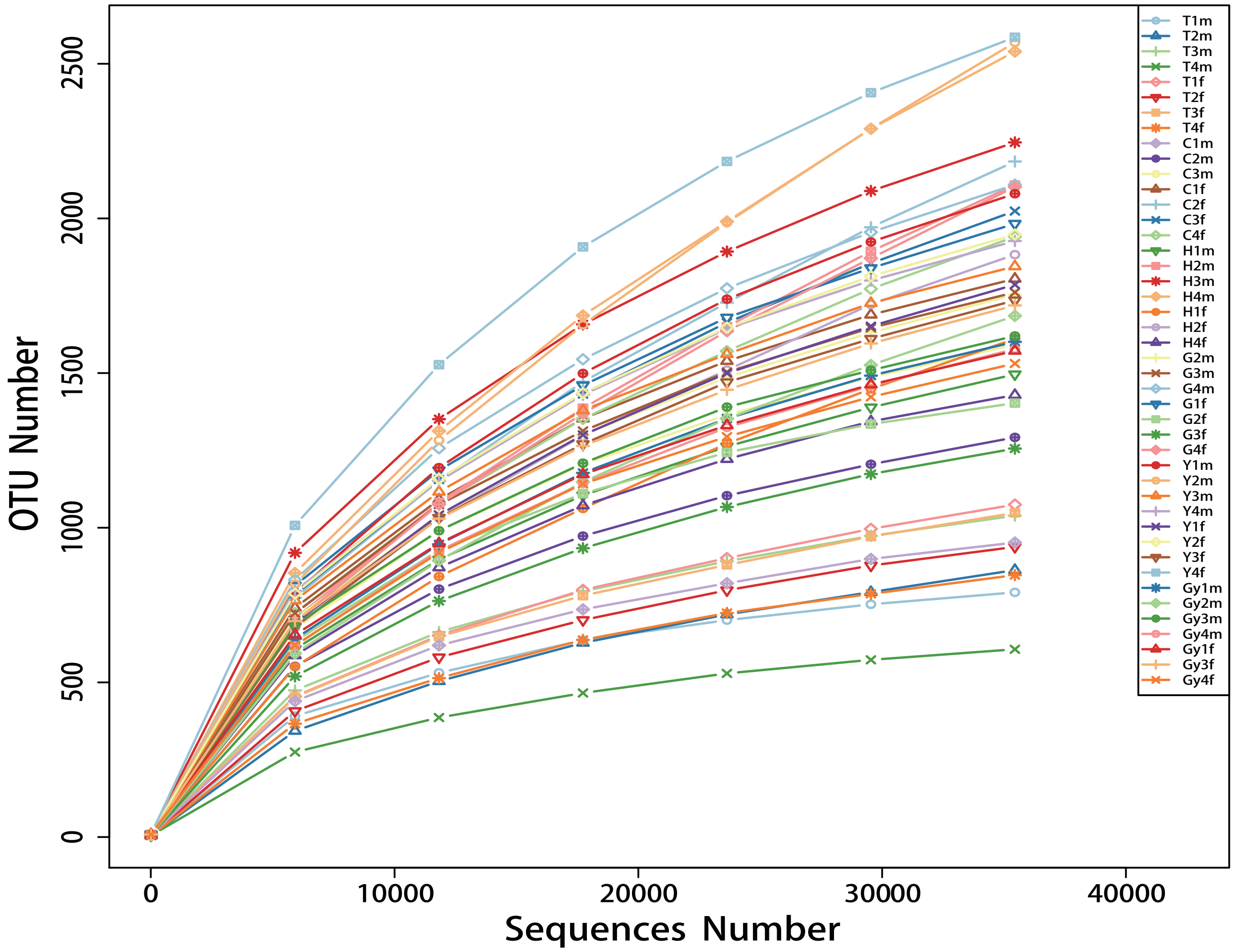

OTU Number
Sequences Number
0
500
1000
1500
2000
2500
0
10000
20000
30000
40000
T1m
T2m
T3m
T4m
T1f
T2f
T3f
T4f
C1m
C2m
C3m
C1f
C2f
C3f
C4f
H1m
H2m
H3m
H4m
H1f
H2f
H4f
G2m
G3m
G4m
G1f
G2f
G3f
G4f
Y1m
Y2m
Y3m
Y4m
Y1f
Y2f
Y3f
Y4f
Gy1m
Gy2m
Gy3m
Gy4m
Gy1f
Gy3f
Gy4f


b

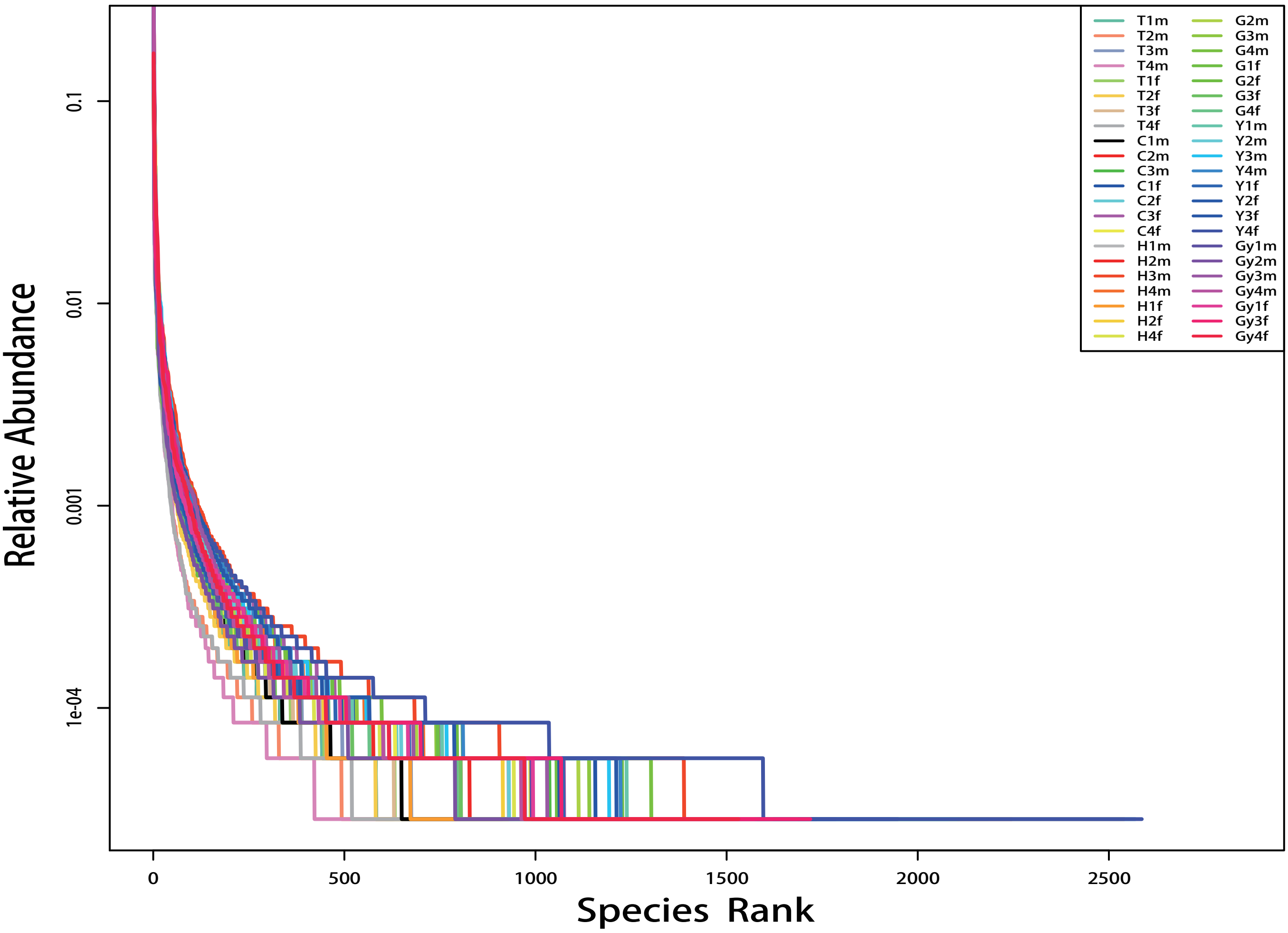

Relative Abundance
Species Rank
0.1
0.01
0.001
1e-04
0
500
1000
1500
2000
2500
T1m
T2m
T3m
T4m
T1f
T2f
T3f
T4f
C1m
C2m
C3m
C1f
C2f
C3f
C4f
H1m
H2m
H3m
H4m
H1f
H2f
H4f
G2m
G3m
G4m
G1f
G2f
G3f
G4f
Y1m
Y2m
Y3m
Y4m
Y1f
Y2f
Y3f
Y4f
Gy1m
Gy2m
Gy3m
Gy4m
Gy1f
Gy3f
Gy4f

**Supplementary Fig. S2.** Dilution and rank abundance curves for all gut bacteria community. **(A)** Rarefaction curves and **(B)** Ranks abundance curves; the x-axis shows the bacterial species and the y-axis shows the relative abundance of the bacteria. Each curve in the graph represents a different crabs and different colors are used to show their lake of origin or group they belong to. As the sequencing depth increased, the number of gut bacteria increased the relative abundance increased in rarefaction curves and decrease in rank abundance curves.
