# Supplementary material for: Relationship Between the Fatty Acid Profiles and Gut Bacterial Communities of the Chinese Mitten Crab (Eriocheir sinensis) From Ecologically Different Habitats
Source: Front Microbiol. 2020 Oct 15;11:565267. doi: 10.3389/fmicb.2020.565267 (PMC7593381; doi:10.3389/fmicb.2020.565267)
Supplement: Supplementary Figure 3 — Core bacterial number among all groups and Shannon index box of six crab populations. (A) Venn diagram showing gut bacteria in each crab population. (B) Box plot of Shannon index showing the differences in the gut bacterial community of the six crab populations. [file Image_3.pdf]

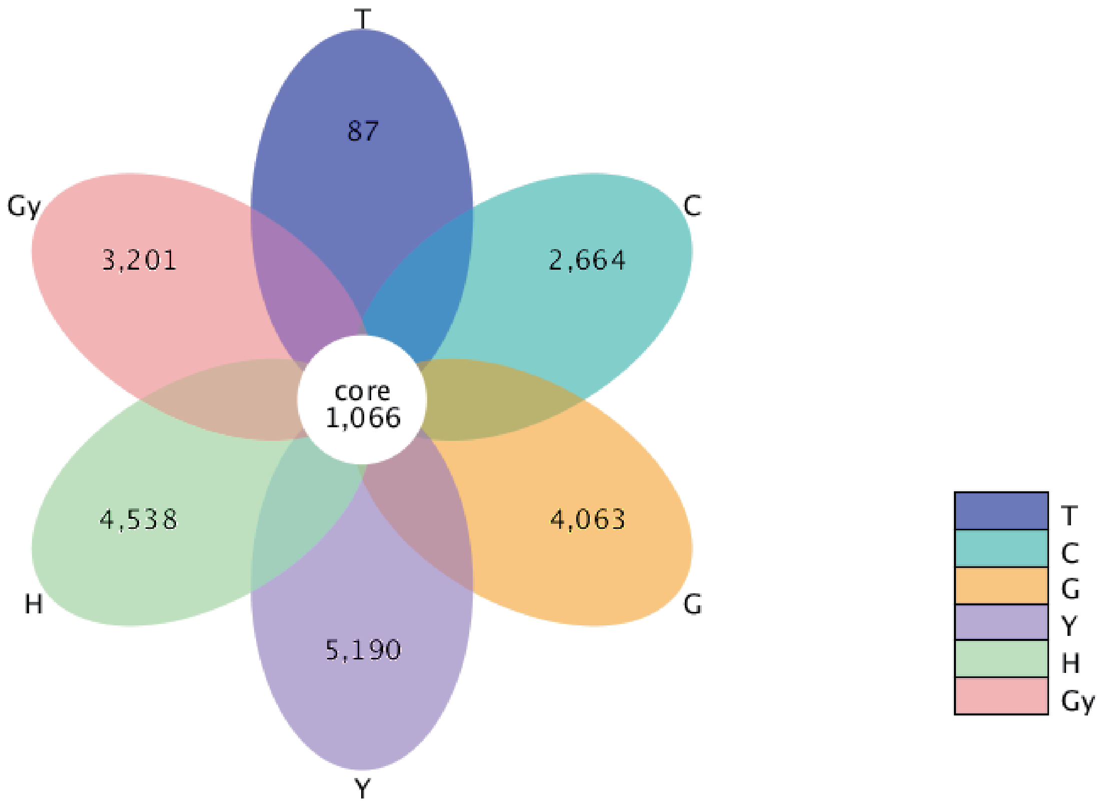
T
C
G
Y
H
Gy
87
2,664
4,063
5,190
4,538
3,201
core
1,066

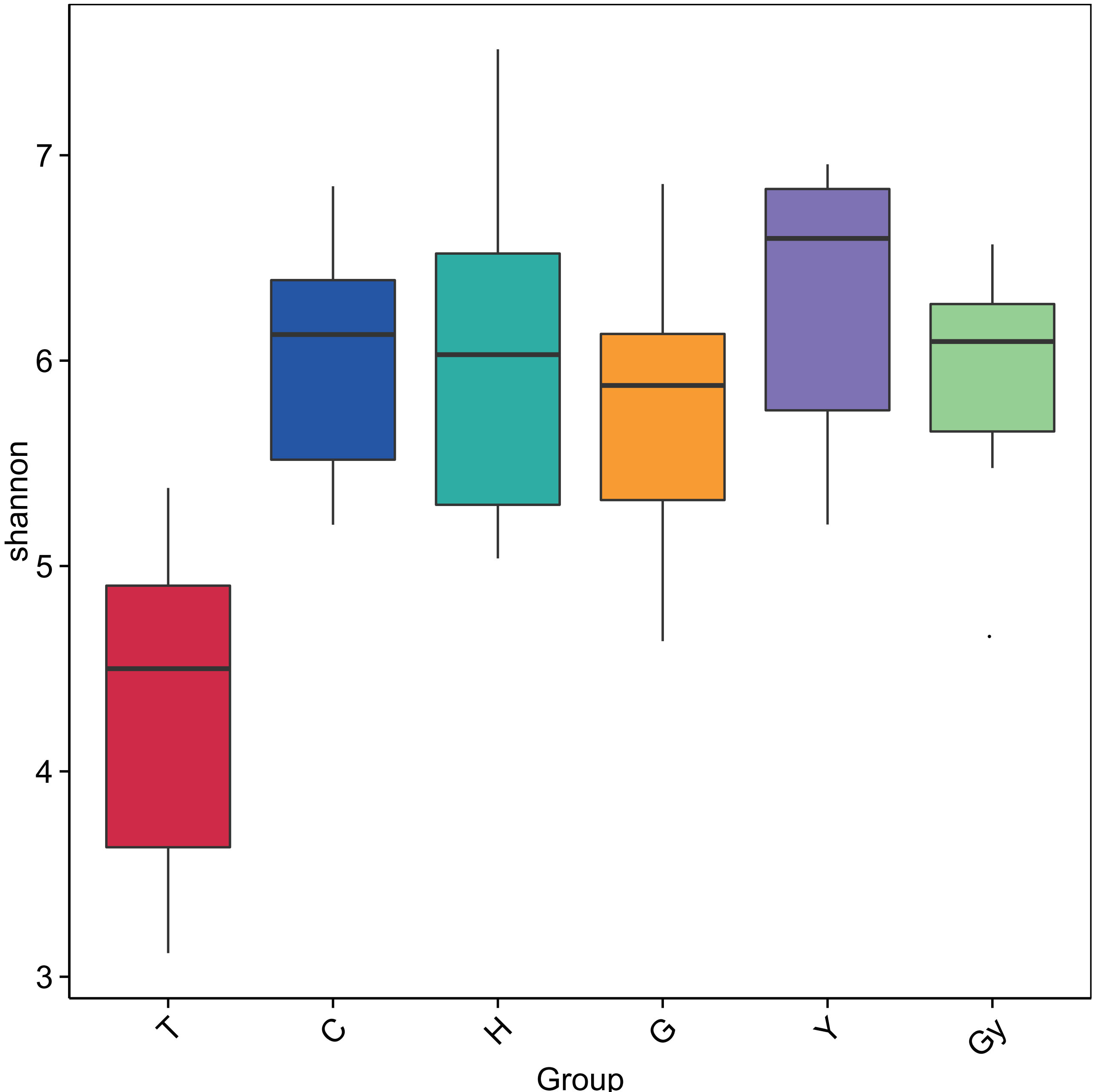
shannon
7
6
5
4
3
T
C
H
G
Y
Gy
Group

Supplementary Fig.S3 Core bacterial number among all groups and Shannon index box of six crab populations. **(A)** Venn diagram showing gut bacteria in each crab population. **(B)** Box plot of Shannon index showing the differences in the gut bacterial community of the six crab populations.
