# Supplementary material for: Relationship Between the Fatty Acid Profiles and Gut Bacterial Communities of the Chinese Mitten Crab (Eriocheir sinensis) From Ecologically Different Habitats
Source: Front Microbiol. 2020 Oct 15;11:565267. doi: 10.3389/fmicb.2020.565267 (PMC7593381; doi:10.3389/fmicb.2020.565267)
Supplement: Supplementary Figure 4 — Gut bacteria species-specific dendrogram of six crab population based on their geographical origin. The pie chart with different colors in the circle consist of crabs from different lakes. The size of each color in the pie is the proportion of the relative abundance of bacteria community from the corresponding lake. The numbers below the taxonomy name represents the average relative abundance percentage of all groups in this level (there are two numbers). [file Image_4.pdf]

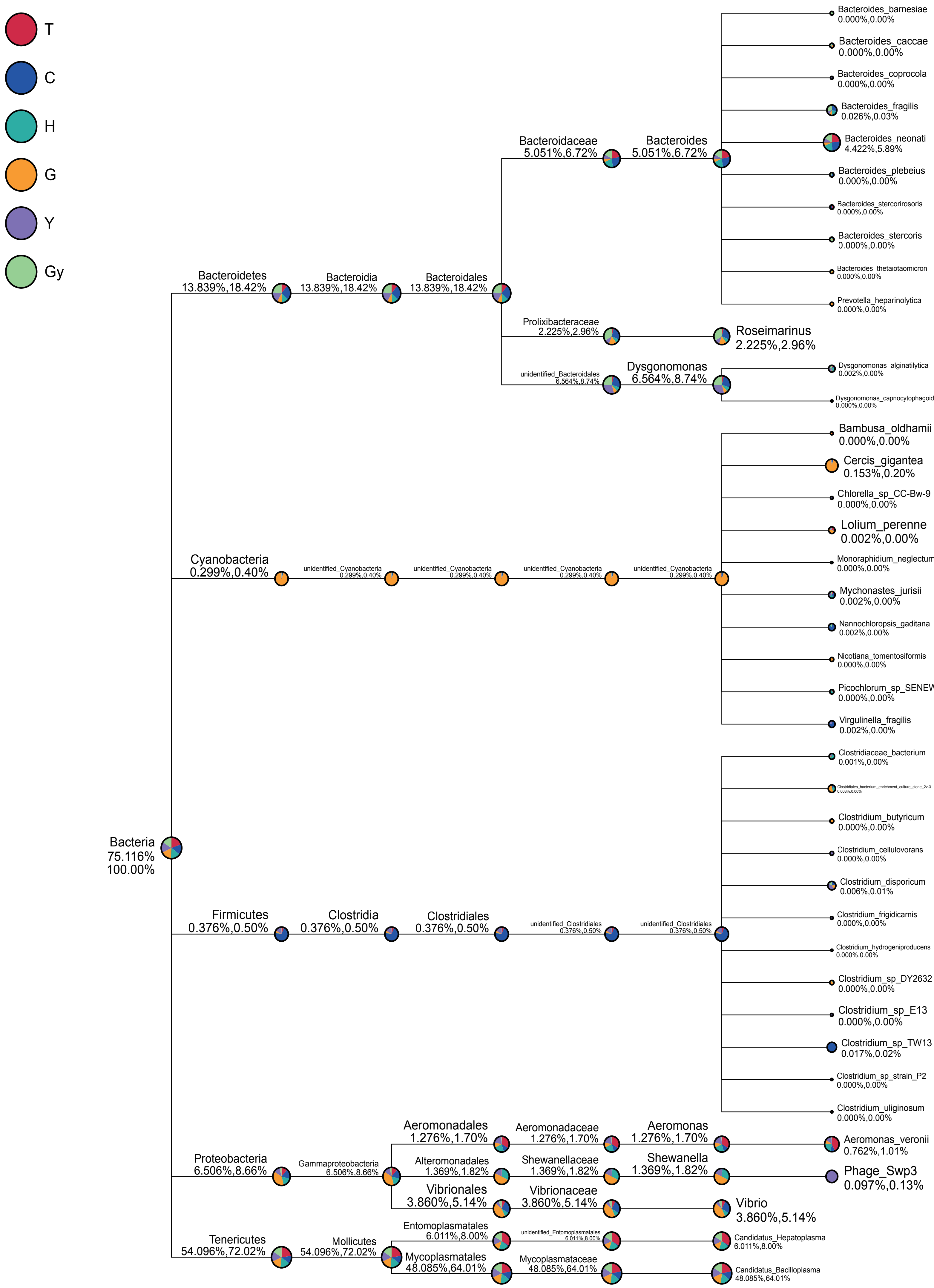

T
C
H
G
Y
Gy
Bacteria
75.116%
100.00%
Bacteroidetes
13.839%,18.42%
Bacteroidia
13.839%,18.42%
Bacteroidales
13.839%,18.42%
Bacteroidaceae
5.051%,6.72%
Bacteroides
5.051%,6.72%
Bacteroides_barnesiae
0.000%,0.00%
Bacteroides_caccae
0.000%,0.00%
Bacteroides_coprocola
0.000%,0.00%
Bacteroides_fragilis
0.026%,0.03%
Bacteroides_neonati
4.422%,5.89%
Bacteroides_plebeius
0.000%,0.00%
Bacteroides_stercorirosoris
0.000%,0.00%
Bacteroides_stercoris
0.000%,0.00%
Bacteroides_thetaiotaomicron
0.000%,0.00%
Prevotella_heparinolytica
0.000%,0.00%
Prolixibacteraceae
2.225%,2.96%
Roseimarinus
2.225%,2.96%
unidentified_Bacteroidales
6.564%,8.74%
Dysgonomonas
6.564%,8.74%
Dysgonomonas_alginatilytica
0.002%,0.00%
Dysgonomonas_capnocytophagoid
0.000%,0.00%
Cyanobacteria
0.299%,0.40%
unidentified_Cyanobacteria
0.299%,0.40%
unidentified_Cyanobacteria
0.299%,0.40%
unidentified_Cyanobacteria
0.299%,0.40%
unidentified_Cyanobacteria
0.299%,0.40%
Bambusa_oldhamii
0.000%,0.00%
Cercis_gigantea
0.153%,0.20%
Chlorella_sp_CC-Bw-9
0.000%,0.00%
Lolium_perenne
0.002%,0.00%
Monoraphidium_neglectum
0.000%,0.00%
Mychonastes_jurisii
0.002%,0.00%
Nannochloropsis_gaditana
0.002%,0.00%
Nicotiana_tomentosiformis
0.000%,0.00%
Picochlorum_sp_SENEW
0.000%,0.00%
Virgulinella_fragilis
0.002%,0.00%
Firmicutes
0.376%,0.50%
Clostridia
0.376%,0.50%
Clostridiales
0.376%,0.50%
unidentified_Clostridiales
0.376%,0.50%
unidentified_Clostridiales
0.376%,0.50%
Clostridiaceae_bacterium
0.001%,0.00%
Clostridiales_bacterium_enrichment_culture_clone_2z-3
0.003%,0.00%
Clostridium_butyricum
0.000%,0.00%
Clostridium_cellulovorans
0.000%,0.00%
Clostridium_disporicum
0.006%,0.01%
Clostridium_frigidicarnis
0.000%,0.00%
Clostridium_hydrogeniproducens
0.000%,0.00%
Clostridium_sp_DY2632
0.000%,0.00%
Clostridium_sp_E13
0.000%,0.00%
Clostridium_sp_TW13
0.017%,0.02%
Clostridium_sp_strain_P2
0.000%,0.00%
Clostridium_uliginosum
0.000%,0.00%
Proteobacteria
6.506%,8.66%
Gammaproteobacteria
6.506%,8.66%
Aeromonadales
1.276%,1.70%
Aeromonadaceae
1.276%,1.70%
Aeromonas
1.276%,1.70%
Aeromonas_veronii
0.762%,1.01%
Alteromonadales
1.369%,1.82%
Shewanellaceae
1.369%,1.82%
Shewanella
1.369%,1.82%
Phage_Swp3
0.097%,0.13%
Vibrionales
3.860%,5.14%
Vibrionaceae
3.860%,5.14%
Vibrio
3.860%,5.14%
Tenericutes
54.096%,72.02%
Mollicutes
54.096%,72.02%
Entomoplasmatales
6.011%,8.00%
unidentified_Entomoplasmatales
6.011%,8.00%
Candidatus_Hepatoplasma
6.011%,8.00%
Mycoplasmatales
48.085%,64.01%
Mycoplasmataceae
48.085%,64.01%
Candidatus_Bacilloplasma
48.085%,64.01%

Supplementary Fig. S4 Gut bacteria species-specific dendrogram of six crab population based on their geographical origin. The pie chart with different colors in the circle consist of crabs from different lakes. The size of each color in the pie is the proportion of the relative abundance of bacteria community from the corresponding lake. The numbers below the taxonomy name represents the average relative abundance percentage of all groups in this level (there are two numbers).
