# Supplementary material for: Relationship Between the Fatty Acid Profiles and Gut Bacterial Communities of the Chinese Mitten Crab (Eriocheir sinensis) From Ecologically Different Habitats
Source: Front Microbiol. 2020 Oct 15;11:565267. doi: 10.3389/fmicb.2020.565267 (PMC7593381; doi:10.3389/fmicb.2020.565267)
Supplement: Supplementary Figure 5 — Cladogram of LEfSe and Heat map of the correlation between gut bacteria and crab’s geographical origin. (A) Cladogram of LEfSe analysis showing abundance of microbial taxa in the six crab populations. The inner circle is the phylogenetic tree constructed by the representative sequences of genera. The color of branches represents their corresponding phylum, each color represents a phylum, and the outer circle represents the relative abundance of each genus in each group. (B) Heat map of the correlation matrix between crab population and the bacteria communities at phylum level. The horizontal axis are the different bacteria phyla represented by different color and on the horizontal axis are the lakes of crab origin. Red orange tones indicate a positive correlation between crab population and the bacteria communities; blue and yellow white indicate absence of correlation as for example. [file Image_5.pdf]

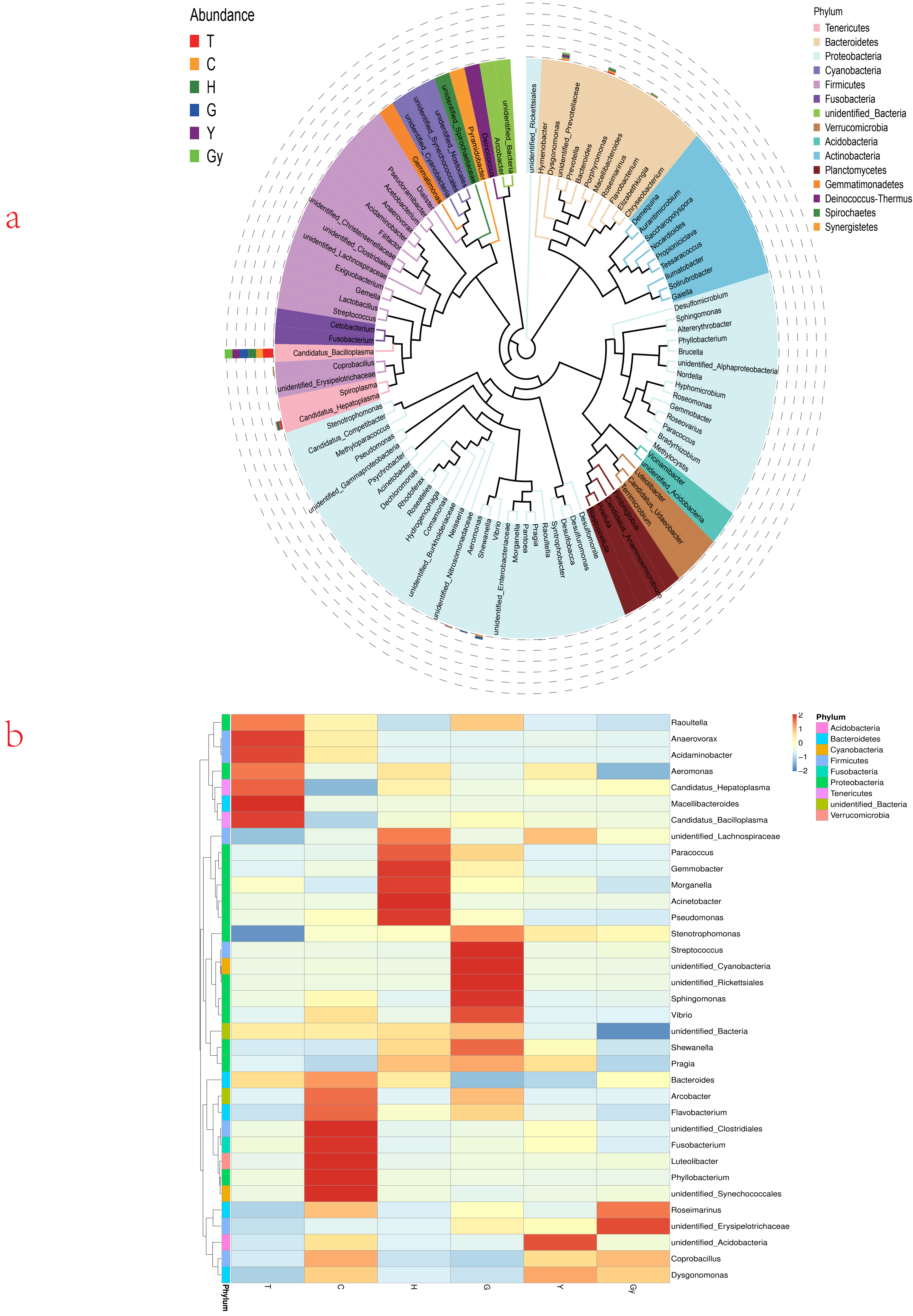
a
Abundance
T
C
H
G
Y
Gy
Phylum
Tenericutes
Bacteroidetes
Proteobacteria
Cyanobacteria
Firmicutes
Fusobacteria
unidentified_Bacteria
Verrucomicrobia
Acidobacteria
Actinobacteria
Planctomycetes
Gemmatimonadetes
Deinococcus-Thermus
Spirochaetes
Synergistetes
unidentified_Rickettsiales
Hymenobacter
Dysgonomonas
unidentified_Prevotellaceae
Prevotella
Bacteroides
Porphyromonas
Macellibacteroides
Roseimarinus
Flavobacterium
Elizabethkingia
Chryseobacterium
Demequina
Aurantimicrobium
Saccharopolyspora
Nocardioides
Propioniciclava
Tessaracoccus
Ilumatobacter
Solirubrobacter
Gaiella
Desulfomicrobium
Sphingomonas
Altererythrobacter
Phyllobacterium
Brucella
unidentified_Alphaproteobacteria
Nordella
Hyphomicrobium
Roseomonas
Gemmobacter
Roseovarius
Paracoccus
Bradyrhizobium
Methylocystis
Vicinamibacter
unidentified_Acidobacteria
Luteolibacter
Candidatus_Udaeobacter
Terrimicrobium
Limnoglobus
Candidatus_Anammoximicrobium
Pirellula
Blastopirellula
Desulfomonile
Desulfuromonas
Desulfobacca
Syntrophobacter
Raoultella
Pragia
Pantoea
Morganella
unidentified_Enterobacteriaceae
Vibrio
Shewanella
Aeromonas
unidentified_Nitrosomonadaceae
Neisseria
unidentified_Burkholderiaceae
Comamonas
Hydrogenophaga
Roseateles
Rhodoferax
Dechloromonas
Acinetobacter
Psychrobacter
unidentified_Gammaproteobacteria
Pseudomonas
Methyloparacoccus
Candidatus_Competibacter
Stenotrophomonas
Candidatus_Hepatoplasma
Spiroplasma
unidentified_Erysipelotrichaceae
Coprobacillus
Candidatus_Bacilloplasma
Fusobacterium
Cetobacterium
Streptococcus
Lactobacillus
Gemella
Exiguobacterium
unidentified_Lachnospiraceae
unidentified_Clostridiales
unidentified_Christensenellaceae
Filifactor
Acidaminobacter
Anaerovorax
Acetobacterium
Pseudoramibacter
Dialister
Gemmatimonas
unidentified_Cyanobacteria
unidentified_Synechococcales
unidentified_Nostocales
unidentified_Spirochaetaceae
Pyramidobacter
Deinococcus
Arcobacter
unidentified_Bacteria
b
2
1
0
−1
−2
Phylum
Acidobacteria
Bacteroidetes
Cyanobacteria
Firmicutes
Fusobacteria
Proteobacteria
Tenericutes
unidentified_Bacteria
Verrucomicrobia
Raoultella
Anaerovorax
Acidaminobacter
Aeromonas
Candidatus_Hepatoplasma
Macellibacteroides
Candidatus_Bacilloplasma
unidentified_Lachnospiraceae
Paracoccus
Gemmobacter
Morganella
Acinetobacter
Pseudomonas
Stenotrophomonas
Streptococcus
unidentified_Cyanobacteria
unidentified_Rickettsiales
Sphingomonas
Vibrio
unidentified_Bacteria
Shewanella
Pragia
Bacteroides
Arcobacter
Flavobacterium
unidentified_Clostridiales
Fusobacterium
Luteolibacter
Phyllobacterium
unidentified_Synechococcales
Roseimarinus
unidentified_Erysipelotrichaceae
unidentified_Acidobacteria
Coprobacillus
Dysgonomonas
Phylum
T
C
H
G
Y
Gy

Supplementary Fig. S5 Cladogram of LEfSe and Heat map of the correlation between gut bacteria and crab's geographical origin. **(A)** Cladogram of LEfSe analysis showing abundance of microbial taxa in the six crab populations. The inner circle is the phylogenetic tree constructed by the representative sequences of genera. The color of branches represents their corresponding phylum, each color represents a phylum, and the outer circle represents the relative abundance of each genus in each group. **(B)** Heat map of the correlation matrix between crab population and the bacteria communities at phylum level. The horizontal axis are the different bacteria phyla represented by different color and on the horizontal axis are the lakes of crab origin. Red orange tones indicate a positive correlation between crab population and the bacteria communities; blue and yellow white indicate absence of correlation as for example.
