# Supplementary material for: Relationship Between the Fatty Acid Profiles and Gut Bacterial Communities of the Chinese Mitten Crab (Eriocheir sinensis) From Ecologically Different Habitats
Source: Front Microbiol. 2020 Oct 15;11:565267. doi: 10.3389/fmicb.2020.565267 (PMC7593381; doi:10.3389/fmicb.2020.565267)
Supplement: Supplementary Figure 6 — LefSe analysis. (A) The cladogram diagram shows the gut bacteria community species with significant differences in the six crab populations at phylum to genus level. The different colors represent different lakes and their corresponding bacteria. (B) Species with significant difference among the different crab populations have LDA score greater than the estimated value; the default score is 4.0. The length of the histogram is equivalent to the LDA score. [file Image_6.pdf]

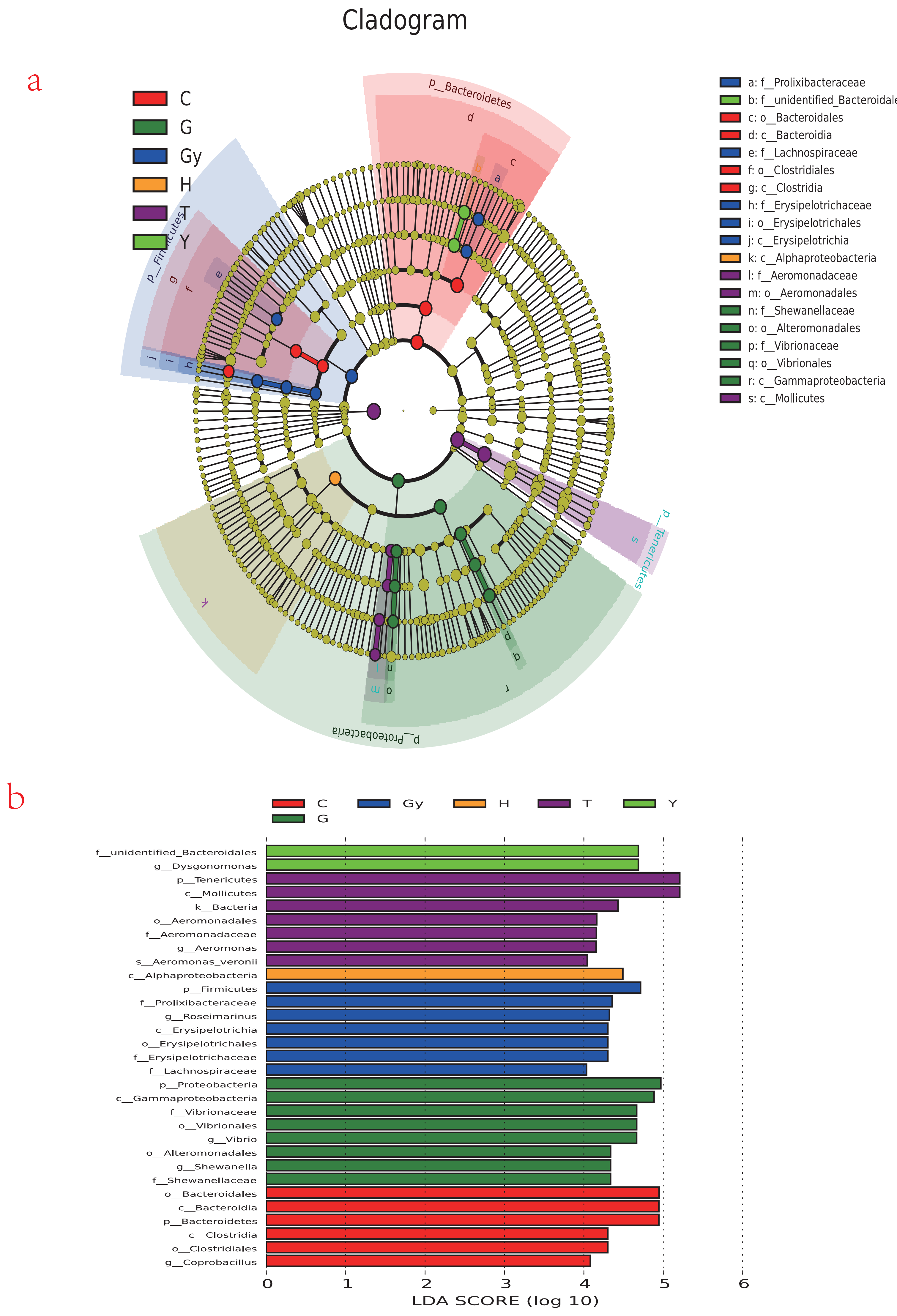
Cladogram
a
C
G
Gy
H
T
Y
p__Bacteroidetes
p__Firmicutes
p__Tenericutes
p__Proteobacteria
a: f__Prolixibacteraceae
b: f__unidentified_Bacteroidale
c: o__Bacteroidales
d: c__Bacteroidia
e: f__Lachnospiraceae
f: o__Clostridiales
g: c__Clostridia
h: f__Erysipelotrichaceae
i: o__Erysipelotrichales
j: c__Erysipelotrichia
k: c__Alphaproteobacteria
l: f__Aeromonadaceae
m: o__Aeromonadales
n: f__Shewanellaceae
o: o__Alteromonadales
p: f__Vibrionaceae
q: o__Vibrionales
r: c__Gammaproteobacteria
s: c__Mollicutes
b
C
Gy
H
T
Y
G
f__unidentified_Bacteroidales
g__Dysgonomonas
p__Tenericutes
c__Mollicutes
k__Bacteria
o__Aeromonadales
f__Aeromonadaceae
g__Aeromonas
s__Aeromonas_veronii
c__Alphaproteobacteria
p__Firmicutes
f__Prolixibacteraceae
g__Roseimarinus
c__Erysipelotrichia
o__Erysipelotrichales
f__Erysipelotrichaceae
f__Lachnospiraceae
p__Proteobacteria
c__Gammaproteobacteria
f__Vibrionaceae
o__Vibrionales
g__Vibrio
o__Alteromonadales
g__Shewanella
f__Shewanellaceae
o__Bacteroidales
c__Bacteroidia
p__Bacteroidetes
c__Clostridia
o__Clostridiales
g__Coprobacillus
0
1
2
3
4
5
6
LDA SCORE (log 10)

Supplementary Fig S6 LefSe analysis. **(a)** The cladogram diagram shows the gut bacteria community species with significant differences in the six crab populations at phylum to genus level. The different colors represent different lakes and their corresponding bacteria. **(B)** Species with significant difference among the different crab populations have LDA score greater than the estimated value; the default score is 4.0. The length of the histogram is equivalent to the LDA score.
